# FAIR, ethical, and coordinated data sharing for COVID-19 response: a scoping review and cross-sectional survey of COVID-19 data sharing platforms and registries

**DOI:** 10.1016/S2589-7500(23)00129-2

**Published:** 2023-09-27

**Authors:** Lauren Maxwell, Priya Shreedhar, Delphine Dauga, Peter McQuilton, Robert F Terry, Alisa Denisiuk, Fruzsina Molnar-Gabor, Abha Saxena, Susanna-Assunta Sansone

**Affiliations:** a Heidelberger Institut für Global Health, Universitätsklinikum Heidelberg, Heidelberg, Germany; b Bioself Communication, Cannes, France; c Oxford e-Research Centre, Department of Engineering Science, University of Oxford, Oxford, UK; d TDR, the Special Programme for Research and Training in Tropical Diseases, WHO, Geneva, Switzerland; e Faculty of Chemistry, Georg-August-Universität Göttingen, Göttingen, Germany; f Heidelberg Academy of Sciences and Humanities, Heidelberg, Germany; g Geneva, Switzerland

## Abstract

Data sharing is central to the rapid translation of research into advances in clinical medicine and public health practice. In the context of COVID-19, there has been a rush to share data marked by an explosion of population-specific and discipline-specific resources for collecting, curating, and disseminating participant-level data. We conducted a scoping review and cross-sectional survey to identify and describe COVID-19-related platforms and registries that harmonise and share participant-level clinical, omics (eg, genomic and metabolomic data), imaging data, and metadata. We assess how these initiatives map to the best practices for the ethical and equitable management of data and the findable, accessible, interoperable, and reusable (FAIR) principles for data resources. We review gaps and redundancies in COVID-19 data-sharing efforts and provide recommendations to build on existing synergies that align with frameworks for effective and equitable data reuse. We identified 44 COVID-19-related registries and 20 platforms from the scoping review. Data-sharing resources were concentrated in high-income countries and siloed by comorbidity, body system, and data type. Resources for harmonising and sharing clinical data were less likely to implement FAIR principles than those sharing omics or imaging data. Our findings are that more data sharing does not equate to better data sharing, and the semantic and technical interoperability of platforms and registries harmonising and sharing COVID-19-related participant-level data needs to improve to facilitate the global collaboration required to address the COVID-19 crisis.

## Introduction

Many public health, ethical, economic, and scientific arguments exist for collecting, harmonising, and sharing public health-related participant-level data from research studies, disease surveillance systems, and routine clinical care.^1^, ^2^ The benefits for data sharing include fast-tracking the development and evaluation of preventive measures, diagnostics, and treatments; avoiding the human and economic cost of unnecessary research; and more effectively distinguishing between clinically relevant and spurious sources of heterogeneity to optimise prevention and treatment measures for diverse populations.^1^, ^2^ The urgency of the COVID-19 pandemic has highlighted the importance of data sharing, sometimes pitting collaborators or similar initiatives against one another in the quest for funding and data producers to support data-sharing activities.

Many researchers share data by uploading their datasets to data lakes or dataverses, data storage, and sharing resources, such as GitHub, Harvard Dataverse, Figshare, Zenodo, Open Science Framework (OSF), Vivli, Dryad, or Mendeley Data, among others. These resources house datasets that are not harmonised at the participant level, and there are few to no restrictions on the types of study designs included in these resources. Although these resources host a large volume of COVID-19 data, a search on March 29, 2023 of GitHub datasets for “COVID-19 data” returned 25 604 results, whereas Zenodo and Harvard Dataverse had more than 10 000 datasets for “COVID-19”—these data lake-type resources require high levels of investment to harmonise participant-level data, and identify and curate study metadata, which complicates cross-dataset analyses.

In this scoping review, we focused on identifying and describing COVID-19-related platforms and registries, which are data sharing resources that conduct prospective or retrospective harmonisation of participant-level health data. In most cases, registries require data contributors to upload data with a shared case report form (CRF),^3^ whereas platforms allow contributors to directly upload datasets with different data dictionaries.^4^, ^5^ Both data sharing platforms and registries might restrict eligibility to certain types of data or populations.^6^ Data sharing platforms generally represent greater investments because of the diverse inputs needed for retrospective harmonisation,^7^ their focus on high-dimensional human or pathogen omics (eg, genomic and metabolomic data) and imaging data types rather than low-dimensional clinical data, and more expansive inclusion criteria, which allow for the collection of a greater volume and diversity of data (see appendix p 2 for working definitions of data sharing resources).

Discovery, access, and provenance and descriptive metadata contain information on dataset creation and licensure, subject area, collection mechanisms, variable definitions, data quality, and are central to the identification of relevant datasets and to their appropriate reuse. Collecting participant-level data and descriptive metadata and harmonising and sharing participant-level data are resource-intensive activities that require expertise in physiology, diagnostics, the trajectory and etiology of infection, risk factors and comorbidities, standards for the interoperability of meta-level data and participant-level data, harmonisation, data sharing-related laws, research ethics, and community engagement.^8^ In addition to concerns about maximising data sharing investments by fostering the interoperability of related platforms and registries,^9^, ^10^ the rush to facilitate COVID-19-related data sharing with the extension of existing platforms and the establishment of novel registries^10^, ^11^ raises several questions related to how data sharing efforts map to the FAIR principles for data resources^12^ and the best practice for ethical reuse of participant-level data.^1^, ^13^

To explore these and other questions, we collected data on a number of domains for evaluating how resources for collecting, harmonising, and sharing participant-level COVID-19 data and related metadata correspond to frameworks for public health-related data sharing, including the Global Research Collaboration for Infectious Disease Preparedness (GloPID-R) Principles of Sharing Data in Public Health Emergencies,^14^ COVID-19 National Core Studies (NCS) Data Sharing Principles,^15^ International Code of Conduct for Data Sharing in Genomic Research,^16^ Global Alliance for Genomics and Health Framework (GA4GH) for responsible sharing of genomic and health-related data,^17^ and the Collective benefit, Authority to control, Responsibility, and Ethics (CARE) Principles for Indigenous Data Governance.^18^

## Methods

We followed the Arksey and O’Malley framework for scoping reviews,^19^ including: (1) prospectively identifying a series of broad research questions in consultation with experts from different fields (eg, ethics, omics, electronic medical record [EMR] data, and FAIR data); (2) developing and iteratively refining the search, study selection, and data extraction fields; (3) a narrative synthesis of results; and (4) consultation with stakeholders to further interpret the results. The scoping review research protocol, registered on the OSF, describes the initial questions, which were refined during the data charting process.

### Survey development and data extraction

We consulted with end users of harmonised, participant-level COVID-19 data from different fields to identify information that would be useful for them to evaluate the utility of different data sharing resources. We developed an online cross-sectional survey to collect the required information with the Research Electronic Data Capture (REDCap)^20^, ^21^ system. Survey domains included general information on the resource (eg, lead organisation, location, and funding), data types collected, linkages between data types at the participant level, resource metrics for success (eg, number of dataset uploads and downloads), criteria used to evaluate resource adherence to the FAIR principles, data access mechanisms and governance structure, data protection processes, ethics review and broad consent-related requirements, community engagement, and benefit sharing with data contributors and source communities. We sent the surveys to resource project management teams and principal investigators that we identified from our review of resource websites and publications. Following regular reminders from September, 2020 up to October, 2022, 20 of the 64 resources we contacted completed the online survey, either partially or fully. When the survey responses contrasted with information available online, we used the survey data.

### Analysis and data charting

We tabulated data from the survey and website review and performed simple statistics to describe patterns across the resources. How FAIRness is evaluated depends on the data type and community-specific needs and preferences. When the FAIR principles were first published,^1^ they were necessarily aspirant and vague. Over time, different interpretations and extensions of the principles have developed, alongside a number of assessment tools.^22^ We conducted a qualitative evaluation of registry and platform adherence to the FAIR principles with four basic criteria: (1) whether the resource was discoverable via a persistent identifier; (2) whether information on how to access data was available on the resource website; (3) whether the resource implemented a community-developed standard for participant-level data or metadata, and (4) whether the resource specified a data usage license or agreement. We considered resources that met none or one of the four criteria as not FAIR and resources that met two or more criteria as FAIR enough.

We also conducted a quantitative evaluation of how registries for harmonising and sharing participant-level clinical data align with the FAIR principles by applying the FAIRshake algorithm^23^ to a set of criteria that we identified as most important for evaluating the utility of these resources (appendix pp 3–6). We adapted existing criteria for our specific use case with a combination of a manual review of the FAIR maturity indicators^24^ and the Research Data Alliance FAIR Data Maturity model output;^25^ and a review of the algorithms used by semi-automated tools, including FAIRshake,^23^ FAIR evaluator,^24^ and FAIR-checker.^26^ The FAIR principles focus on the machine-actionability of data and related metadata, findability, accessibility, interoperability, and reusability.^1^ The quantification of how registries for harmonising and sharing participant-level clinical data map to the FAIR criteria is presented in the appendix (pp 7–9) with the important caveat that quantitative evaluations are at an exploratory stage. The research community continues to harmonise the algorithms used to evaluate the application of FAIR indicators across disciplines, as many tools for quantifying FAIRness yield divergent results. Therefore, we focus our discussion on the results of the qualitative evaluation of FAIRness, according to the four main criteria described earlier. Lastly, we reviewed how the registry or platform practices corresponded to best practice defined by the aforementioned data sharing principles and the CoreTrustSeal (CTS) certification for trusted repositories.^27^ CTS launched in 2017 and evolved from the Data Seal of Approval and the International Council for Science World Data System certification frameworks. CTS is meant to help researchers navigate the ecosystem of data reuse resources by evaluating whether they align with 16 core criteria related to technological and organisational infrastructure, data and metadata management and curation, governance, security, and interoperability.^27^ All figures were created in Tableau Online 2022.4 and Flourish.^28^

## Results

We identified 44 registries and 20 platforms that collected, harmonised, and sometimes shared participant-level COVID-19 human subjects’ data (table 1). All but six resources were identified from monthly Google searches rather than natural language processing (NLP) approaches (appendix pp 12–20). We excluded 13 COVID-19-specific resources that did not harmonise participant-level data, did not include data from more than one hospital or system, or did not have a functional website at the time of data collection.

**Table 1:** Overview of platforms and registries for collecting, harmonising, and sharing participant-level COVID-19-related data

	**Pre-dated COVID-19**	**Funding source**	**Population-specific restrictions**	**Data types (linkage to clinical data)**	**Observational or intervention data**	**Longitudinal or cross-sectional data**	**Community-developed standards for metadata or participant-level data**	**COVID-19 status**	**Harmonisation**	**Governance (controllers of access to the data)**	**Who can access the data**	Metrics of success*
**Registries**												
American College of Radiology COVID-19 Imaging Research Registry (USA)	No	Professional organisation	Patients of all ages; with imaging examinations; in USA	Clinical, imaging (linked)	Observational	Longitudinal	Yes	Infected	Retrospective	DAC	Any group with an approved application	No
American College of Surgeons COVID-19 Registry (USA)	No	NS	Patients aged ≥18 years; admitted to the hospital; any location	Clinical	Observational	Longitudinal	No	Infected	Prospective	NS	NS	No
American Society for Hematology Research Collaborative COVID-19 Registry for Hematologic Malignancy (USA)	No	Donation	Patients of all ages across the world with haematological conditions	Clinical	Observational	Cross-sectional	No	Infected	Prospective and retrospective	NA†	NA†	Number of cases
American Society of Clinical Oncology Survey on COVID-19 in Oncology Registry (USA)	No	Foundation	Patients of all ages with cancer; in USA	Clinical	Observational	Longitudinal	Yes	Infected	Prospective	DAC	Any group with an approved application	Number of cases
British Association of Dermatologists Biologic and Immunomodulators Register (University of Manchester, UK)	Yes	Industry	People of all ages with psoriasis; in UK and Ireland	Clinical	Observational and intervention	Longitudinal	Yes	Infected and non-infected	Prospective	NS	Any group with an approved application	Number of participating centres
Cardiac Complications in Patients with SARS Corona virus 2 registry (University Medical Center Utrecht, Netherlands)	No	NS	Patients aged ≥18 years; any location with cardiovascular complications	Clinical	Observational	Longitudinal	Yes	Infected	Prospective	DAC	Any group with an approved application	Number of cases
Center for International Blood and Marrow Transplant Research COVID-19 Data Collection (USA)	Yes	Industry	Patients of all ages who are autologous; allogeneic haematopoietic cell transplant recipients; any location	Clinical	Observational	Longitudinal	No	Infected	Prospective	DAC and resource management team	In most cases the registry will run requested analysis; however, in rare instances individual participant-level data will be shared as well	Number of cases
Coronavirus and MS Reporting Database (Washington University, USA)	No	Professional organisation	Patients of all ages; with CNS demyelinating diseases; in North America	Clinical	Observational	Cross-sectional	No	Infected	Prospective	NA†	NA†	Number of cases
American Heart Association COVID-19 CVD Registry (USA)	No	Professional organisation, donation	Patients aged ≥18 years; with CVD; in USA	Clinical	Observational	Cross-sectional	No	Infected	Prospective and retrospective	DAC	Any group with an approved application	Number of cases
COVID-19 Dermatology Registry (Massachusetts General Hospital, USA)	No	Professional organisation	Patients of all ages; with dermatological manifestations associated with or before COVID-19 infection; any location	Clinical	Observational	Cross-sectional	No	Infected	Prospective	DAC	Any group with an approved application	No
COVID-19 Global Pediatric Rheumatology Database (Boston Children's Hospital, USA)	No	Foundation	Paediatric patients aged ≤18 years with rheumatic disease; any location (except EU)	Clinical	Observational	Cross-sectional	No	Infected	Prospective	DAC	Any group with an approved application	Number of cases
COVID-19 Registry (Rice University, USA)	No	University	People aged ≥18 years; in USA	Clinical	Observational	Longitudinal	No	Infected and non-infected	Prospective	Resource management team	Any group with an approved application	Number of cases
COVID-Hepatology Registry (Translational Gastroenterology Unit, Oxford University, UK)	No	Government, university, professional organisation	Patients of all ages; with liver disease or liver transplant; any location (except the Americas, China, Japan, North and South Korea, and Mongolia)	Clinical	Observational	Cross-sectional	No	Infected	Prospective	DAC	Any group with an approved application	Number of cases
Discovery Viral Infection and Respiratory Illness Universal Study COVID-19 Registry (Society of Critical Care Medicine, USA)	No	NGO	Patients of all ages; admitted to the hospital or intensive care unit; any location	Clinical	Observational	Longitudinal	Yes	Infected	Prospective	DAC	Only groups that contributed data or are otherwise part of the consortium	Number of cases
European Academy of Neurology Neuro-covid Registry (Austria)	No	Professional organisation	Patients aged ≥18 years; with neurological conditions; any location	Clinical	Observational	Longitudinal	No	Infected	Prospective	Resource management team	Only groups that contributed data or are otherwise part of the consortium	No
Extracorporeal Life Support Organization Registry (USA)	Yes	Professional organisation, donation	Patients aged ≥16 years; on extracorporeal membrane oxygenation; any location	Clinical	Observational	Longitudinal	Yes	Infected	Prospective	DAC	Only groups that contributed data or are otherwise part of the consortium	Number of cases
Global Hidradenitis Suppurativa COVID-19 Registry (University of California, San Francisco, USA)	No	NS	Patients of all ages; with hidradenitis suppurativa; any location	Clinical	Observational	Cross-sectional	No	Infected	Prospective	NA†	NA†	Number of cases
Global Registry of COVID-19 in Pediatric Cancer (St. Jude Children's Research Hospital, USA)	No	University, professional organisation	Patients aged ≤18 years; with cancer; any location	Clinical	Observational	Longitudinal	No	Infected	Prospective	NA†	NA†	Number of cases
Global Registry of COVID-19-related Diabetes (King's College London, UK, and Monash University, Australia)	No	University	Patients of all ages; with new-onset diabetes or acute complication of pre-existing diabetes; any location	Clinical	Observational	Longitudinal	No	Infected	Prospective	NA†	NA†	Number of cases
Health Outcome Predictive Evaluation for COVID 19 (St. Carlos Hospital, Spain)	No	NS	Patients of all ages who have been discharged (deceased or alive) from any hospital since September, 2020; any location	Clinical	Observational	Longitudinal	No	Infected	Prospective	NS	Only groups that contributed data or are otherwise part of the consortium	Number of participating centres
International COVID-19 and Pregnancy Registry (Centre HospitalierUniversitaire Vaudois, Switzerland)	No	University	Pregnant women not considered minors; any location	Clinical	Observational	Longitudinal	No	Infected	Prospective	Resource management team	Any group with an approved application	No
Lean European Open Survey for SARS-CoV-2 Infected Patients (University Hospital of Cologne and Goethe University Frankfurt, Germany)	No	Professional organisation	Patients of all ages; any location	Clinical	Observational	Cross-sectional	Yes	Infected	Prospective	DAC and resource management team	Any group with an approved application	Number of cases
Pediatric COVID-19 Case Registry (St Jude Children's Research Hospital, USA)	No	No funding	Patients aged <21 years; in USA	Clinical	Observational	Longitudinal	No	Infected	Prospective	DAC and resource management team	Any group with an approved application	Number of cases
Pregnancy Coronavirus Outcomes Registry (University of California, San Francisco Women's Health Clinical Research Center, USA)	No	University, private donations	Pregnant or recently pregnant women aged ≥13 years; in USA	Clinical	Observational	Longitudinal	No	Infected	Prospective	NA†	NA†	Number of cases
Psoriasis Patient Registry for Outcomes, Therapy and Epidemiology of COVID-19 Infection (Guy's and St Thomas' Hospital, UK)	No	Professional organisation, university	Patients of all ages; with psoriasis; any location	Clinical	Observational	Cross-sectional	No	Infected	Prospective	DAC	Any group with an approved application (except for commercial organisations)	Number of cases
SECURE-Alopecia (National and International Skin Registry Solutions, Ireland)	No	NGO	Patients of all ages; with alopecia; any location	Clinical	Observational	Cross-sectional	No	Infected	Prospective	NA†	NA†	Number of cases
SECURE-Atopic Dermatitis (National and International Skin Registry Solutions, Ireland)	No	NGO	Patients of all ages; with atopic dermatitis; any location	Clinical	Observational	Cross-sectional	No	Infected and non-infected	Prospective	NA†	NA†	Number of cases
SECURE-Celiac (Columbia University, USA)	No	Industry	Patients of all ages; with coeliac disease; any location	Clinical	Observational	Cross-sectional	No	Infected	Prospective	NA†	NA†	Number of cases
SECURE-Eosinophilic Esophagitis and Eosinophilic Gastrointestinal Diseases (Schneider Children's Medical Center in Israel, Israel)	No	NS	Patients of all ages; with eosinophilic gastrointestinal diseases; any location	Clinical	Observational	Cross-sectional	No	Infected	Prospective	NS	Any group with an approved application	Number of cases
SECURE-Inflammatory Bowel Disease (University of North Carolina at Chapel Hill, USA)	No	Industry	Patients of all ages; with inflammatory bowel disease; any location	Clinical	Observational	Cross-sectional	No	Infected	Prospective	DAC and resource management team	Any group with an approved application	Number of cases
SECURE-Liver (University of North Carolina at Chapel Hill)	No	Professional organisation	Patients of all ages; with chronic liver disease or post-liver transplant; in North and South America, China, Japan, and North and South Korea	Clinical	Observational	Cross-sectional	No	Infected	Prospective	NA†	NA†	Number of cases
SECURE-Psoriasis (Wake Forest School of Medicine, USA)	No	NS	Patients of all ages; with psoriasis; any location	Clinical	Observational	Cross-sectional	No	Infected	Prospective	NA†	NA†	Number of cases
SECURE-Sickle Cell Disease (edical College of Wisconsin, USA)	No	NS	Patients of all ages; with sickle cell disease; any location	Clinical	Observational	Cross-sectional	No	Infected	Prospective	NA†	NA†	Number of cases
SECURE-vascular anomalies (Children's Hospital of Philadelphia, USA)	No	NS	Patients of all ages; with vascular anomalies; any location	Clinical	Observational	Cross-sectional	No	Infected	Prospective	NA†	NA†	Number of cases
Society for Cardiovascular Magnetic Resonance COVID-19 Registry (USA)	No	NS	Patients of all ages; with cardiovascular complications and cardiovascular magnetic resonance data; any location	Clinical; imaging (linked)	Observational	Longitudinal	Yes	Infected	Prospective	DAC and data generator	Any group with an approved application	Number of participating centres
Society of Vascular and Interven. Neurology COVID-19 Registry (Cooper University Hospital, USA)	No	Professional organisation	Patients aged ≥18 years; with cerebrovascular complications; in USA, Spain, Egypt, and Romania	Clinical	Observational	Longitudinal	No	Infected	Prospective	Resource management team	Any group with an approved application	Number of participating centres
Surveillance of COVID-19 in Patients with T1D (T1D Exchange, USA)	No	Professional organisation, NGO, industry	Patients of all ages; with type 1 diabetes; in USA	Clinical	Observational	Longitudinal	No	Infected	Prospective	NA†	NA†	Number of data contributors
The COVID-19 and Cancer Consortium (Vanderbilt University Medical Center, USA)	No	NS	Patients aged ≥18 years; with cancer; in USA, EU, Argentina, Canada, Mexico, and UK	Clinical	Observational	Longitudinal	Yes	Infected	Prospective	NA†	NA†	Number of cases
The COVID-19 Global Rheumatology Alliance Registry (University of California, San Francisco, USA)	No	Industry	Patients aged >18 years; with rheumatic disease; any location (except EU)	Clinical	Observational	Cross-sectional	No	Infected	Prospective	DAC and resource management team	Only groups that contributed data or are otherwise part of the consortium	Number of cases
The European Alliance of Associations for Rheumatology COVID-19 Registry (Switzerland)	No	Professional organisation	Patients of all ages; with rheumatic disease; in EU	Clinical	Observational	Cross-sectional	No	Infected	Prospective	DAC and resource management team	Only groups that contributed data or are otherwise part of the consortium	Number of cases
The European Renal Association COVID-19 Database (University Medical Center Groningen, Netherlands)	No	Professional organisation, industry	Patients aged ≥18 years; with kidney disease; in EU countries bordering the Mediterranean region	Clinical	Observational	Longitudinal	No	Infected	Prospective	NA‡	NA‡	Number of cases
The UK Coronavirus Cancer Monitoring Project (University of Birmingham, UK)	No	University	Patients of all ages; with cancer; in UK	Clinical	Observational	Longitudinal	No	Infected	Prospective	NS	NS	Number of participating centres
The UK Paediatric Oncology Coronavirus Cancer Monitoring Project (University of Birmingham, UK)	No	University	Patients aged <16 years; with cancer; in UK	Clinical	Observational	Longitudinal	Yes	Infected	Prospective	NS	NS	Number of cases
Thoracic Cancers International COVID-19 Collaboration Registry (Fondazione Istituto di Ricovero e Cura a Carattere Scientifico Istituto Nazionale Tumori, Vanderbilt Uni. Medical Center, Italy)	No	Professional organisation, university	Patients of all ages; with thoracic cancer; any location	Clinical	Observational	Longitudinal	No	Infected	Prospective	NS	NS	Number of cases
**Platforms**												
Canadian COVID-19 Genomics Network–HostSeq Portal (Genome Canada, Canada)	No	Government	Patients of all ages; in Canada	Clinical; human omics (linkage not specified)	NS	NS	Yes	Infected	Prospective and retrospective	DAC	NA§	NA§
Canadian COVID-19 Genomics Network - VirusSeq Data Portal (Genome Canada, Canada)	No	Government	Patients of all ages; in Canada	Clinical; pathogen omics (linked)	Observational	Cross-sectional	Yes	Infected	Prespecified standard	Open access	Open access	Number of viral genome sequences
China National GeneBank DataBase (China)	Yes	Government	Patients of all ages; any location	Pathogen omics (unlinked)	Observational	Cross-sectional	Yes	Infected	Prespecified standard	Public data is open access; requests for controlled data overseen by reviewers or data owning organisation	Public data, open access; controlled data, any group with an approved application; private data, not accessible	No
Consortium for Clinical Characterization of COVID-19 by EHR (Harvard Medical School, USA)	No	Professional organisation, private foundation	Patients of all ages; any location	Clinical	Observational	Both	Yes	Infected	Retrospective	NS	NS	No
COVID-19 and MS—a global data sharing initiative (Multiple Sclerosis Data Alliance, Belgium)	No	Professional organisation, industry	Patients of all ages; with multiple sclerosis; any location	Clinical	Observational and interventional	Both	No	Infected	Prospective and retrospective	NA†	NA†	Number of cases
DNA Data Bank of Japan (Research Organization of Information and Systems, National Institute of Genetics, Japan)	Yes	Government	People of all ages; any location	Human omics (unlinked); pathogen omics (unlinked)	Observational	Cross-sectional	Yes	Infected and non-infected	Prespecified standard	Data generator	Open access	No
Electron Microscopy Data Bank (EMBL-EBI, UK)	Yes	Government	People of all ages; any location	Imaging (unlinked)	Observational	Cross-sectional	Yes	Infected and non-infected	Prespecified standard	Open access	Open access	Number of SARS-CoV-2 electron cryo-microscopy maps
Electron Microscopy Public Image Archive (EMBL-EBI, UK)	Yes	Government	People of all ages; any location	Imaging (unlinked)	Observational	Cross-sectional	Yes	Infected and non-infected	Prespecified standard	Open access	Open access	Number of entries for SARS-CoV-2 imaging data
European Genome-Phenome Archive (EMBL-EBI, UK)	Yes	Government	People of all ages; any location	Clinical; human omics (linked); pathogen omics (linked); imaging (linked)	Observational and interventional	Cross-sectional	Yes	Infected and non-infected	Prespecified standard	DAC	Any group with an approved application	Number of dataset results for COVID-19
European Nucleotide Archive (EMBL-EBI, UK)	Yes	Government	People of all ages; any location	Human omics (unlinked); pathogen omics (unlinked)	Observational	Cross-sectional	Yes	Infected and non-infected	Prespecified standard	Open access to public data; data generator can restrict data access	Public data, open access; controlled data, any group with an approved application; private data, not accessible	Number of sequence results for COVID-19
GenBank (NCBI-NLM, USA)	Yes	Government	People of all ages; any location	Human omics (unlinked); pathogen omics (unlinked)	Observational and interventional	Cross-sectional	Yes	Infected and non-infected	Prespecified standard	Open access	Open access	Number of SRA runs and nucleotide records for SARS-CoV-2
Gene Expression Omnibus (NCBI-NLM, USA)	Yes	Government	People of all ages; any location	Human omics (unlinked); pathogen omics (unlinked)	Observational and interventional	Cross-sectional	Yes	Infected and non-infected	Prespecified standard	Open access	Open access	RNA and SRA sample results for SARS-CoV-2
GeneWeaver (The Jackson laboratory, Baylor University, and The University of Tennessee, USA)	Yes	Government	People of all ages; any location	Human omics (unlinked)	Observational and interventional	Both	Yes	Infected and non-infected	Prespecified standard	Open access to public data; data generator can restrict data access	Public data, open access; controlled data, any group with an approved application; private data, not accessible	Human gene set results for COVID-19 and for SARS-CoV-2
Global Initiative on Sharing All Influenza Data (GISAID) (Freunde von GISAID e.V., Germany)	Yes	Government	Patients of all ages; with influenza or coronavirus infection; any location	Pathogen omics (unlinked)	Observational	Cross-sectional	Yes	Infected	Prespecified standard	Resource management team	Any group with an approved application	Number of human COVID-19 genome sequence submissions
Infectious Diseases Data Observatory (Oxford University, UK)	Yes	Government	Patients of all ages; any location; with emerging pathogens or neglected diseases	Clinical	Observational and interventional	Longitudinal	Yes	Infected	Prospective and retrospective	DAC	Any group with an approved application	Number of observations and patients
International COVID-19 Data Alliance (Health Data Research UK, UK)	No	NGO, industry, private foundation	Patients of all ages; any location	Clinical	Observational and interventional	Both	No	Infected and non-infected	Each project has different data types, some standardized to dictionaries, others did not	Resource management team or DAC depending on dataset and nature of request	Only groups that contributed data or are otherwise part of the consortium	Number of datasets
National Institute of Health–National COVID Cohort Collaborative (National Center for Data to Health by National Center for Advancing Translational Sciences hub sites, USA)	No	Government	Patients of all ages; in USA	Clinical; human omics (linked); pathogen omics (linked); imaging (linked)	Observational	Longitudinal	Yes	Infected and non-infected	Prospective	DAC	Any group with an approved application	Number of cases
QMENTA imaging database (QMENTA, USA and Spain)	Yes	Industry	Patients of all ages; with imaging exam data; any location	Clinical; imaging (linked)	Observational	Cross-sectional	Yes	Infected and non-infected	Any format	Data generator	Registered users invited to access the images by the image uploaders	Number of COVID-19 images
The database of Genotypes and Phenotypes (NCBI-NLM, USA)	Yes	Government	People of all ages; any location	Clinical; human omics (linked); pathogen omics (linked)	NS	Both	Yes	Infected and non-infected	Prespecified standard	DAC	Any group with an approved application	Number of phenotype dataset and molecular dataset results for COVID-19
The Immunology Database and Analysis Portal (University of California, San Francisco, USA; Stanford University, USA; University of Buffalo, USA; and Technion–Israel Institute of Technology, and Northrop Grumman, Israel)	Yes	Government	Patients of all ages; any location	Clinical; human omics (linked); pathogen omics (linked)	Observational and interventional	Both	Yes	Infected and non-infected	Prespecified standard	Data generator	Any group with an approved application	Number of studies and subjects for COVID-19

CVD=cardiovascular disease. DAC=data access committee. EBI=European Bioinformatics Institute. EMBL=European Molecular Biology Laboratory. NA=not applicable. NCBI=National Center for Biotechnology Information. NGO=non-governmental organisation. NLM=The National Library of Medicine. NS=not specified. SECURE=Surveillance Epidemiology of Coronavirus Under Research Exclusion. SRA=sequence read archive. T1D=type 1 diabetes.

* Based on data obtained from the resources' websites in September, 2022.

† Participant-level data will not be shared.

‡ Participant-level data will not be shared, but resource will conduct requested analyses.

§ Data are not yet available (platform is not launched even though data collection has started, or resource is not yet accepting data).

### Data type-specific resources

COVID-19 data sharing resources were overwhelmingly data type-specific. Almost all registries (42 of 44; 95%) were restricted to clinical data and two (5%) registries included clinical and high-dimensional imaging data. Ten (50%) of the 20 platforms included human omics data, 11 (55%) included pathogen omics data, and four (20%) included high-dimensional imaging data (eg, CT scans). 11 (55%) platforms included more than one data type.

Registry-specific and platform-specific linkages between data types at the participant-level are described in the appendix (p 21). Approximately a third of platforms (seven; 35%) and more than half of the registries (24; 55%) included longitudinal clinical data. Although no registries included human or pathogen omics data, four (20%) platforms included longitudinal human omics data, one of which also included longitudinal imaging data. Two platforms included linked longitudinal clinical and human and pathogen omics data. Another platform included longitudinal data of all data types, including clinical, host and pathogen omics, and high-dimensional imaging data.

### Population, comorbidity, or body system-specific resources

Most registries (42 of 44; 95%), but few platforms (two of 20; 10%) restricted data to populations with a particular co-infection, comorbidity, assessment, treatment, or outcome of interest. There were several instances of registries that covered the same comorbidities, including six (14%) for different forms of cancer, six (14%) for skin conditions, four (9%) for blood conditions, three (7%) related to cardiovascular system diseases, three (7%) for rheumatic disease, three (7%) for issues related to the digestive system, two (5%) for liver disease, two (5%) for neurological conditions, and two (5%) for diabetes. An additional two (5%) registries were restricted to individuals with kidney disease and patients receiving extracorporeal membrane oxygenation. Several registries collected data on paediatric (four; 9%) or pregnant (two; 5%) populations. Of the paediatric-focused registries, two registries included data on paediatric cancer patients and one on paediatric patients with rheumatic disease. All platforms and 28 (64%) registries included data from participants of all ages. Nine (20%) registries were restricted to data for adults aged 18 and over.

The global distribution of platforms and registries for harmonising and sharing COVID-19 participant-level data are shown in the appendix (pp 22–23). Around one-third of platforms (six of 20; 30%) and 27 (61%) of 44 registries were based in the USA; two (10%) platforms, and ten (23%) registries were based in Europe; six (30%) platforms and five (11%) registries were based in the UK; one registry was based in Israel, and another in both England and Australia. Two platforms (10%) were based in Canada, one in China, one in Japan, one in the USA and Spain, and another in the USA and Israel. 16 (80%) platforms and 25 (57%) registries accepted data from any country; four (20%) platforms and 19 (43%) registries were country-specific or region-specific.

### Database management and funding

Almost all of the registries (40 of 44; 91%) but few platforms (three of 20; 15%) were related to professional organisations, such as the American Heart Association, American Society of Clinical Oncology, International League of Dermatological Societies, the COVID-19 Global Rheumatology Alliance, and the Extracorporeal Life Support Organization who were engaged in the creation of the registry and its dissemination to the organisation's network. Nine (21%) registries and three (15%) platforms received funding from more than one source. 16 platforms (80%) and one registry received government funding; three (15%) platforms and 19 (43%) registries received funding from related professional organisations or non-governmental organisations (NGOs); and three (15%) platforms and seven (16%) registries received funding from industry sponsors. Some registries (ten; 23%) and none of the platforms received funding from universities. Other registries (six; 14%) and platforms (two; 10%) received funding from private donations or foundations.

### Harmonisation of participant-level data

For the 44 registries and 11 platforms that collected clinical data, most registries (41; 93%) and one platform were restricted to prospective harmonisation of participant-level data via a shared case report form (CRF). Three of the 11 (27%) platforms that collected clinical data and two (5%) registries conducted both prospective and retrospective harmonisation, whereas one clinical data registry and one platform conducted only retrospective harmonisation. Of the 39 registries that included prospective harmonisation of clinical data via an electronic-CRF (eCRF), 28 (72%) provided a REDCap-based eCRF. Among other data capture software, registries also used Qualtrics (two; 5%), OpenApp (two; 5%), or SurveyMonkey (one; 2%); one platform that conducted both prospective and retrospective harmonisation of clinical data used QMENTA, and the Infectious Diseases Data Observatory (IDDO) platform allowed data entry either with REDCap (prospective harmonisation) or its own platform (retrospective harmonisation). At the beginning of the COVID-19 pandemic, WHO, International Severe Acute Respiratory and Emerging Infection Consortium (ISARIC), and IDDO created an open access series of REDCap-based eCRFs,^29^ which applied Clinical Data Interchange Standards Consortium's (CDISC) Study Data Tabulation Model (SDTM) standards.^30^ Of the 55 resources that included clinical data, only one platform (IDDO itself) and one registry (Cardiac Complications in Patients with SARS Corona Virus 2 Registry) reported using the joint WHO, ISARIC, and IDDO eCRFs, which represents a missed opportunity for prospective harmonisation for COVID-19 response.

### Implementation of FAIR principles

We present an overview of how COVID-19-related resources for collecting, harmonising, and sharing participant-level data map to the FAIR principles and best practice for ethical and equitable data sharing (table 2; appendix pp 24–26 for related text from each set of principles). Platforms were generally more closely aligned with the FAIR principles than registries that were comorbidity-specific or population-specific (figure). Additionally, platforms and registries harmonising clinical or epidemiological data were much less FAIR than those that harmonised high-dimensional data types. The registry and platform names corresponding to the figure can be found in the appendix (pp 27–31).

**Table 2:** How COVID-19-related data sharing efforts map to established principles for data sharing

	**How COVID-19 data sharing resources correspond**	**Seven GloPID-R principles of sharing data in public health emergencies**^14^	**COVID-19 NCS data Sharing principles**^15^	**International code of conduct for data sharing in genomic research**^16^	**GA4GH framework for responsible sharing of genomic and health-related data**^17^	**CARE principles for Indigenous data governance**^18^
Collaboration	Six platforms and two registries had explicit connections between clinical–epidemiological and other data types at the participant-level; registry data were siloed by comorbidity, body system, and population	..	Yes	Yes	Yes	..
FAIR data	Nine platforms and five registries met the four basic criteria for FAIRness; no platforms and ten registries met none of the criteria	Yes	Yes	Yes	..	..
Ethical	Two platforms and three registries were restricted to data that included broad consent for future use or waiver of consent; eight platforms and 20 registries only shared de-identified data; three platforms and five registries did not seek ERC approval (of those that specifically mentioned approval-related information); six registries and 11 platforms required or suggested citation of data providers in publications; 24 registries and one platform required or suggested acknowledgement of data providers or co-authorship on related publications	Yes	Yes	Yes	Yes	Yes
Community engagement	Three platforms and ten registries mentioned community engagement; two platforms and 17 registries included a data dashboard	Yes	Yes	Yes	..	Yes
Transparent governance	Eight platforms and no registries were open access or had some data that were open access; six platforms and 17 registries had a DAC; data providers decided data access for six platforms and one registry; seven registries and one platform did not specify how data access was mediated	Yes	Yes	Yes	Yes	..
Compliance with data protection laws	Not assessed	..	Yes	Yes	Yes	..
Evaluate platform utility	17 platforms and 39 registries provided some measure of resource utility	..	Yes	..	Yes	Yes
Quality	Not assessed	Yes	..	Yes	Yes	..
Timely	As of February 2023, participant-level data were available for 17 platforms and 24 registries	Yes	..	..	..	..

CARE=Collective Benefit, Authority to Control, Responsibility, Ethics. DAC=data access committee. ERC=ethics review committee. FAIR=finable, accessible, interoperable, reusable. GA4GH=Global Alliance for Genomics and Health. GloPID-R=Global Health Security Initiative and Global Research Collaboration for Infectious Disease Preparedness. NCS=National Core Studies.

**Figure:**
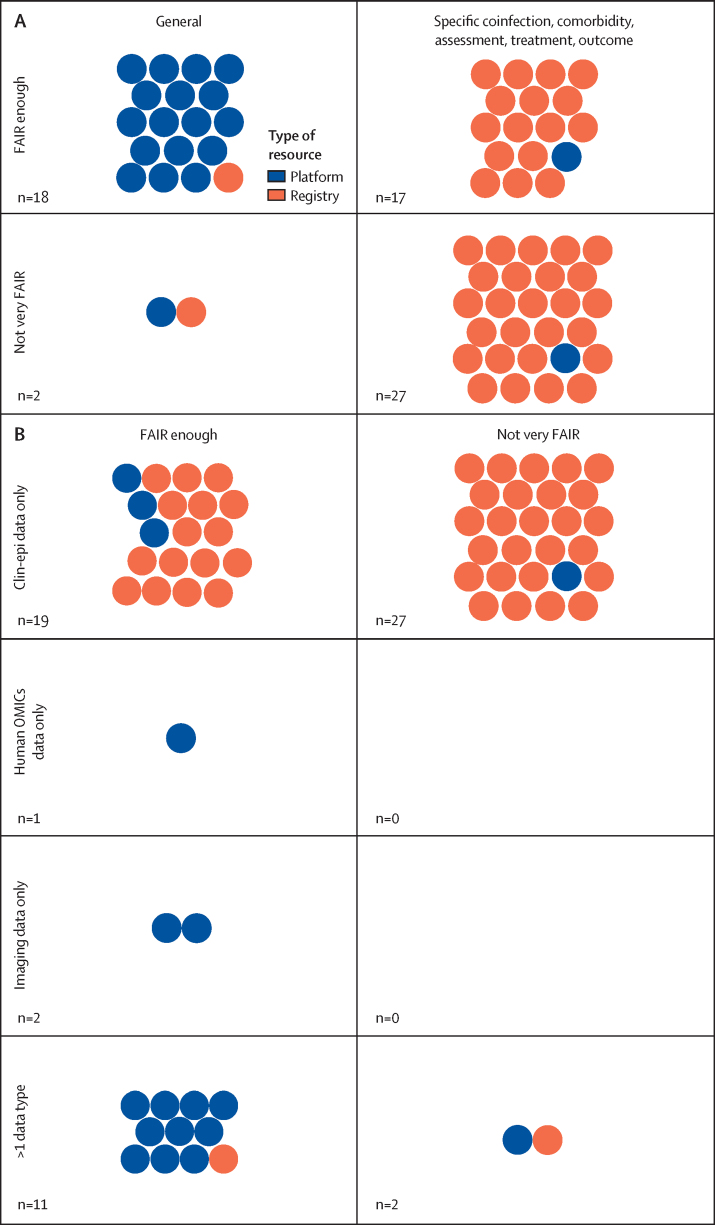
Qualitative evaluation of FAIRness (A) Disease-specific platform and registry correspondence with the FAIR criteria for data resources. (B) Participant-level data types hosted by platforms and registries, and correspondence with the FAIR criteria for data resources.

For our qualitative assessment of FAIRness, nine (45%) of 20 platforms and five (11%) of 44 registries met all four of the criteria for FAIRness, whereas no platforms and ten (23%) registries met none of the criteria. Platforms that met all of the criteria for FAIRness were large, government-funded platforms that pre-existed COVID-19. The five registries that met all four criteria for FAIRness collected clinical data only, harmonised this data prospectively, and had data access committees (DACs) in place to oversee the use of data. Registries that met none of the criteria for FAIRness were generally COVID-19-specific resources that are still accepting data and might develop in terms of infrastructure and governance for COVID-19 data collection and sharing in the future. The application of a community-developed standard for participant-level data or metadata was the most commonly missed component of FAIRness, only four (36%) of 11 platforms and eight (18%) of 44 registries that collected participant-level clinical data had adopted a community-developed data capture or exchange standard. Our evaluation, which included the registration and curation of eligible resources identified from our search in FAIRsharing.org, improved the FAIRness of a number of platforms and registries by (1) the assignment of a digital object identifier; (2) the collection and publication of machine readable, discovery metadata, including data on resource content and data types, and resource implementation of data and metadata standards; and information on data accessibility mechanisms and terms of use. As part of this Review, a digital object identifier, which improves resource findability, was assigned to two (10%) of 20 platforms and 18 (41%) of 44 registries, which previously did not have a persistent identifier.

Community-developed standards for data and metadata capture and exchange, which include minimal information reporting requirements, terminologies, and models and formats, are needed to structure the data in an unambiguous manner for humans and machines.^31^ Standards are more clearly defined and widely used for high-dimensional data types when machine-readable metadata are defined as part of the data capture (eg, Digital Imaging and Communications in Medicine standards for imaging data) than for clinical data, which then relates to the comparable FAIRness of resources for harmonising and sharing omics and imaging data versus resources for sharing clinical or epidemiological data. Of the 44 clinical data registries, eight (18%) used an eCRF that mapped to internationally accepted standards for clinical data. Three registries used International Classification of Diseases-10 codes. Other data capture standards for clinical data that were each used by one registry included CDISC standards, Systematized Nomenclature of Medicine (SNOMED) Clinical Terms, Critical Care Data Dictionary, Medical Dictionary for Regulatory Activities, Current Procedural Terminology codes, Unified Medical Language System controlled unique identifier, National Cancer Institute thesaurus, and Anatomical Therapeutic Chemical classification. All of the 12 platforms that currently share omics data mapped to internationally accepted metadata or participant-level data standards. Two followed Minimum Information About a Microarray Experiment standards.

The use of shared community-developed data capture standards for participant-level data and metadata facilitates cross-resource analyses. The appendix (pp 32–34) shows standards-based and technical interoperability between the COVID-19 data sharing resources; all resources, registries, and platforms are shown separately. Interoperability between data sharing resources is facilitated by (1) terminology artefacts, including controlled vocabularies and ontologies, which facilitate semantic interoperability and are shown as maroon dots in the figure; (2) models and formats, including transmission formats, which define the structure and relationship of information are shown in dark blue; and (3) reporting guidelines, including minimum information requirements and checklists that provide information needed to contextualise the digital object and are shown in green. We were not able to identify standards used by many registries, which means that data hosted by these registries will not be interoperable with comparable data hosted by other registries.

Of the platforms, one explicitly stated that participant-level data would not be shared, one intended to share data at a later time, and one other platform did not specify any information regarding sharing participant-level data. On the other hand, 16 (36%) of 44 registries did not intend to share participant-level data, and four (9%) did not specify information regarding data sharing. Three (13%) of the 24 registries and one of the 18 platforms that explicitly stated their intention to share data did not provide any information on how to access data on their website. Six (25%) registries that intended to share data provided insufficient information on how to access data on their website. 16 (67%) of the 24 registries and seven (39%) of the 18 platforms that intended to share data did not have a data usage license or agreement mentioned on their website.

### Governance

The protection of human subjects, the governance of and mechanism for data sharing, and engaging in meaningful benefit sharing with the research team that contributed data and the participants’ source community are of central importance for ethical data sharing.^2^ Of the ten platforms that shared or intended to share human omics data, two were open access, three included both open access data and restricted access to sensitive data moderated by the data generators, four required DAC permission to access the data, and the remaining platform required data generator permission to access the data. 17 of the 24 registries and six of the nine platforms that shared or intended to share clinical data, four of the ten platforms that shared or intended to share human omics data, and the two platforms that shared or intended to share linked clinical and human or pathogen omics or imaging data, included a DAC to review data requests. Requests for data access for six (30%) of the 18 platforms and one (4%) of the 24 registries that shared or intended to share data were decided by the data contributors.

### Ethical concerns

Close to half of the registries (20 [45%] of 44) stated that they were exempt from ethics review committee (ERC) oversight because they only collected de-identified data. Although nine (45%) of 20 platforms only collected de-identified data, only one claimed ERC exemption. Two (10%) platforms and three (7%) registries stated that they would only accept participant-level data from groups that included broad consent for future use in their informed consent forms or have obtained a waiver of consent.

Benefit sharing with the community that participated in research and with groups that contribute data is an important part of ethical data reuse. 17 (39%) registries and two (10%) platforms planned to disseminate or were disseminating aggregate findings with a data dashboard. Furthermore, 18 (90%) of the platforms and 30 (68%) of the registries mentioned other forms of benefit sharing, including citation of the groups that provided data (11 platforms and six registries), citation of the data sharing resource itself (17 platforms and 11 registries), acknowledgment of data providers or co-authorship on related publications (24 registries and one platform), or access to analytic tools (eight platforms and one registry). Only ten (23%) registries and three (15%) platforms mentioned any form of community engagement. Community engagement activities included community forums to guide the overall direction of the platform, involving patient representatives in the management of the registry, work with health-care providers to understand the implications for clinical practice, and active engagement with researchers and their communities in low-income and middle-income countries (LMICs) with training and dissemination activities.

### Metrics for resource utility

There are no clearly defined metrics for determining whether a platform or registry is successful or not. Data sharing resources reported the number of collaborating centres, datasets, participants represented by those datasets, and SARS-CoV-2 genome sequences, registered users, and views or downloads of datasets to describe the breadth of data collection and dissemination work. Five (17%) of the 29 registries and two (11%) of the 18 platforms that were accepting data did not include any information that could be used to characterise data submission or reuse.

### Adherence to best practices for data management, access, and preservation

CTS is an international certification for data repositories that was developed to help researchers and funders understand how repository or platforms adhere to best practice in data management, preservation, and governance.^27^ One of the platforms (ImmPort)^32^ and none of the registries were CTS certified. To be eligible for CTS certification, the mission of data resources should state their intention to preserve and provide access to data. We reviewed the mission statements of registries and platforms and found eight (40%) of 20 platforms and two (5%) of 44 registries stated their intention to preserve and provide access to data. During the final revisions of this paper, one platform and three registries that no longer had functioning websites were classified as not intending to provide access to and preserve data.

## Discussion

In this Review, we present the results of a major initiative by members of the COVID-19 Clinical Research Coalition to understand how participant-level data are being shared for COVID-19 response. In addition to monthly searches on Google and Google Scholar, we applied NLP to the COVID-19 Open Research Dataset and consulted with colleagues that work on sharing human or pathogen omics data or clinical data in Europe, Canada, Africa, Latin America, and Asia to identify resources for collecting, harmonising, and sharing COVID-19-related participant-level data. We identified 64 platforms and registries for collecting, harmonising, and sometimes sharing different types of COVID-19 data. For close to a third of these resources, information to evaluate resource FAIRness or governance practices were collected from our online survey. Although we expect that these responses would still be up-to-date, existing resources have continued to evolve and additional data sharing registries or platforms that harmonise participant-level data have emerged since we completed our search in June, 2021. Because the relevant data from platforms and registries are generally not machine readable, continuously updating and curating the results requires a substantial investment of time. When we updated and reran our search and reconducted our screening between October, 2021 and 2022, we found an additional 103 COVID-19-related registries or platforms. Almost half (43 of 103) of the new platforms and registries included a general adult population either having had COVID-19 (active or past) or having been vaccinated against it. The other half included a range of comorbidities or population-related restrictions. For example, 14 of the new registries included data on COVID-19 in pregnant women and another four registries included data on COVID-19 in individuals younger than 21 years. Eight registries were restricted to individuals with cancer and COVID-19 and four to those with cardiovascular or haematological conditions and COVID-19. Collecting the detailed data needed to describe these additional resources was not possible for this Review. The additional registries and platforms identified in our later search do not change the substantive findings of this report and reinforce the need to coordinate, rather than duplicate, related initiatives to improve data access. Language bias in the search for resources, despite the use of NLP to address this bias and the continuing evolution of platforms and registries are considerable limitations of this Review.

### How do COVID-19 data-sharing resources correspond to existing data-sharing principles?

Although the importance of leveraging existing participant-level data and of connecting different data types at the participant level for COVID-19 response cannot be overstated, more resources for data sharing does not mean better data sharing. The GloPID-R Principles of Sharing Data in Public Health Emergencies,^14^ International Code of Conduct for Data Sharing in Genomic Research,^16^ GA4GH Framework for responsible sharing of genomic and health-related data,^17^ and CARE Principles for Indigenous Data Governance^18^ were established before the NCS Data Sharing Principles^15^ were published in response to the COVID-19 pandemic. We review how COVID-19 data sharing platforms and registries map to the cross-framework principles of collaboration; adherence to the FAIR principles; ethical issues, including transparent governance, protection of sensitive data, and community engagement; compliance with data protection laws; and evaluation of platform utility. The actual text for each principle from each framework is presented in the appendix (pp 24–26). The correspondence of data sharing resources to established principles is summarised in table 2. Commonly shared challenges and recommendations for coordinated data sharing for COVID-19 response are presented in panel 1; stakeholder-specific recommendations are summarised in panel 2.

Panel 1 **Commonly shared challenges and recommendations for coordinated data sharing for COVID-19 response**

**Challenge: interoperability and accessibility of electronic medical record (EMR) and sensitive research data**

Recommendations: expand efforts to link EMRs by shared standards (eg, Fast Healthcare Interoperability Resources Health Level 7 international patient summary or Observational Medical Outcomes Partnership common data models [CDM]). Make EMR and sensitive research data (eg, linked human omics and clinical data) accessible via shielded platform-based approaches that allow analysing data without moving the data (eg, DataSHIELD). Promote interoperability-based data reuse by promoting standards and providing open access codes for commonly applied analyses (eg, Observational Health Data Sciences and Informatics).

**Challenge: interoperability and reuse of EMR and research data**

Recommendations: apply standards to observational research that are closely related to or the same as EMR data standards (eg, Systematized Nomenclature of Medicine or Logical Observation Identifiers Names and Codes). Consider ethical imperative to use EMR data for improving health care.

**Challenge: resources siloed by data type, comorbidity, and body system**

Recommendations: address the root causes of data silos; for example, a lack of interoperability or siloed data at the data generating group level, concerns about legal implications, and vendor reluctance to share data or re-identification concerns.

**Challenge: interoperability of platforms and registries**

Recommendations: develop open access tools and guidance for meta harmonisation across standards. Provide open access trainings on application of CDM-based approaches.

**Challenge: interoperability of governance structures**

Recommendations: develop guidance on the best practice for platform and registry governance. Develop more sensitive methods of exploring the possibility of re-identification.

**Challenge: resources have different degrees of FAIRness (findable, accessible, interoperable, reusable)**

Recommendations: register your resources in a system like FAIRsharing to become more discoverable, indicate which data and metadata standards you implement, describe your data accessibility mechanisms, and declare terms of use for your data. Provide support to users. Maximise connections with other resources.

**Challenge: benefit sharing and community engagement**

Recommendations: develop guidance for the best practices for community engagement. Provide support and foster accountability.

**Challenge: competition between data sharing resources**

Recommendations: incentivise cooperation. Address technical barriers to inter-resource interoperability. Develop metrics for assessing the utility of data sharing platforms and registries. Develop guidance for the best practice for platform governance and hold platforms and registries to those standards.

Panel 2 **Recommended actions for stakeholders to support coordinated data sharing efforts for COVID-19 and beyond**

**Stakeholder: funders**

Recommendations: develop and implement metrics to quantify the return on investment in data sharing efforts. Take concrete steps to make data more FAIR (findable, accessible, interoperable, and reusable); for example, recommend that resources register in a system with machine readable metadata like FAIRsharing.org. Require prospective registration of observational studies in a repository that collects metadata and assigns a digital object identifier (DOI). Require a proportion of the budget to be put towards interoperability (eg, implementing community-developed standards for participant-level data). Require intervention and observational research studies to apply community-developed standards. Support metacatalogues which facilitate data reuse by helping researchers obtain DOIs (eg, FAIRsharing.org).

**Stakeholder: journal editors**

Recommendations: require a DOI to be assigned to a participant-level dataset and research protocol to improve study and data discoverability. Require implementation of a machine-readable FAIR checklist that covers issues related to data availability, interoperability, and registration of metadata. Incentivise data reuse.

**Stakeholder: regulators**

Recommendation: create a regulatory body for observational research.

**Stakeholder: bioinformaticians, software developers, data stewards, and the open science community**

Recommendations: conduct mixed methods research to understand where and when datasets are made available, including barriers and facilitators to using platforms with different governance structures. Build connections between data sharing infrastructures so that when data are uploaded to one platform they are also automatically available on other platforms. Expand open science initiatives to facilitate data reuse without data access; for example, shielded approaches to data access, access on open source code, and interoperable participant-level data and metadata. Conduct mixed methods research to understand where and when datasets are made available, including barriers and facilitators to using platforms with different governance structures. Implement in tools, curation processes, recommendations, and guidelines, the use of community-defined descriptive standards to enable structured reporting and meaningful reuse of data and metadata. Refine and pilot specific indicators for evaluating the FAIRness of clinical and epidemiological data. Foster compliance with best practices for governance related to the future use of data or samples that is consistent with international ethics guidelines on the topic.

**Stakeholder: legal**

Recommendations: address real or perceived data protection law barriers to data access, particularly regarding general data protection regulation, with cross-national governance and legislation, and clarification of interpretation and the application of existing laws. Identify and address provincial, state, and governorate-level legal barriers with regards to margins of implementation and interpretation. Identify and address legal barriers related to reuse of data for various secondary purposes, including dependence of the primary purpose. Identify and address legal barriers related to the reuse of data from protected minority groups under the perspectives of fairness and equity. Identify misinterpretations of data protection roles (controller, processor, joint controller, and subprocessor). Clarify the connection between actors' data protection roles and their role in defining how data are used; work on modalities of involving data submitting communities, entities, and actors into decisions about secondary data usage. Work towards data protection governance that allows data participants to assert their rights also in international data sharing contexts. Clarify legal tools for international data transfers in emergency situations, such as pandemics. Define technical and data security measures necessary to protect international data transfers in emergency situations and, if no established legal tool for the transfer has been defined, to offer data protection, but also to allow data processing and interpretation. Elaborate collision rules when legal frameworks interact across national boundaries.

**Stakeholder: ethics advisory bodies**

Recommendations: raise awareness of health-care providers, researchers, and other stakeholders about ethics guidelines for data sharing, data reuse, and reuse of medical data for research purposes. Strengthen guidelines on privacy and confidentiality (and their limitations) within the scope of data reuse and data sharing. Work across regulatory and legal entities and stakeholder groups to harmonise guidelines, and ensure consistency of approaches in the interpretation of shared ethical concerns. Provide community-developed recommendations for community engagement related to different types of data sharing or data reuse-related infrastructures. Provide community-developed recommendations on governance for different types of data sharing or data reuse-related infrastructures. Require a section on FAIR data as part of ethics submissions for observational research.

**Stakeholder: data sharing platforms or registries**

Recommendations: build expertise in related community-developed standards. Meaningfully engage communities on data sharing. Evaluate understanding of language around broad consent for future use.

### Data siloed by data type and comorbidities

The siloing of data by type, comorbidity, and treatment increases the time required for sharing data when individuals with multiple comorbidities need to be entered into various databases and ultimately diminishes the utility of the data. The existing universe of disease-specific or treatment-specific registries might lead to the exclusion of important populations affected by multiple comorbidities.

Post-COVID-19 condition (also known as long COVID) affects approximately 30–87% of adults^33^, ^34^ and 5–8% of children^35^, ^36^ and harmonised, longitudinal datasets with linked clinical, human and pathogen omics, and imaging data might facilitate post COVID-19 condition-related prognosis and treatment. Furthermore, this data can help identify participant-level factors correlating to the emergence of variants of concern (VOC) and VOC-related differences in cause and vaccine efficacy. Only a few of the 64 resources included clinical data linked to human and pathogen omics data at the participant level, which hinders efforts to respond to emerging and established VOCs.

### eCRFs and other efforts at facilitating prospective harmonisation of participant-level COVID-19 data

In contrast to previous epidemics of emerging pathogens, the partnership between IDDO, ISARIC, and WHO resulted in the rapid publication of a series of REDCap-based eCRFs that apply CDISC SDTM standards.^37^, ^38^ Other than IDDO itself, only one of the 55 data sharing resources that collect COVID-19-related clinical data reported accessing the joint IDDO, ISARIC, or WHO eCRFs, which represents a missed opportunity. In addition to these eCRFs, there have been a number of national efforts to facilitate the interoperability of COVID-19 data, including the US National Coordinator for Health Information Technology Logica COVID-19 Implementation Guide,^39^ which applies a Health Level 7 Fast Healthcare Interoperability Resources-based library of COVID-19-related data elements, and the UK National Health Service COVID-19 National Clinical Coding Standards.^40^ International efforts, such as the COVID-19 Interoperability Alliance,^41^ which addresses cross-national interoperability of COVID-19 data with SNOMED, Logical Observation Identifiers Names and Codes (LOINC) and RxNorm, the Health Level 7 International Patient Summary Implementation Guide,^42^ and the European Health Data Space initiatives^43^ have emerged to address cross-national interoperability of EMR data.

### The need for connections between research and clinical data streams

Selection bias (when the participants included in a study or database differ systematically from the population of interest)^44^ is an important consideration when accessing data uploaded to the platforms and registries described here. EMR data are an underutilised resource for surveillance and epidemic response^45^, ^46^ and represent a less selected population than those reflected in data that are manually entered by hospital staff in disease-specific registries. Only one platform, 4CE, included EMR data. Formidable barriers, including a lack of interoperability, ethical concerns, and EMR-vendor or hospital-specific barriers to access,^47^ have prevented coordinated sharing of EMR data and might have led to the current universe of comorbidity-specific and population-specific registries. In addition to reducing the data entry burden incurred when data are shared by registries rather than EMR, there are compelling ethical arguments, including the duty of easy rescue, for using EMR data in the public health response to epidemics^48^ and several ongoing initiatives to facilitate cross-national, interoperable EMR data.^43^, ^49^, ^50^, ^51^

### FAIR principles and community-developed data capture or transfer standards

Our results show that platforms for sharing high-dimensional participant-level omics and imaging data align better with FAIR principles than registries for sharing clinical or epidemiological data. This difference is explained, in part, by the inclusion of machine-readable metadata and community-developed standards for participant-level data as part of the computational processing of high-dimensional data, discipline-specific expectations regarding data availability, the use of community-developed standards, and limited regulatory oversight for observational health research. Registries for participant-level clinical data were less likely to be assessed for their adherence to the FAIR principles before the COVID-19 pandemic, and how to measure the FAIRness of clinical or epidemiological data is an actively evolving conversation. The FAIR principles focus on machine readability and use and reuse of data at scale; they do not address the quality and utility of the data resource and its content. The FAIR community continues to work towards finalising cross-disciplinary, cross-data type maturity indicators that can be implemented by any evaluation tool to yield consistent results. Our evaluation of resource adherence to the FAIR principles should be read in the context of this evolving landscape. Funders can build on this initial evaluation of clinical research data sharing efforts by bringing together stakeholders and disciplines to develop indicators to benchmark COVID-19 data sharing initiatives towards FAIR data.

Around a quarter of the 64 COVID-19-focused platforms and registries met none of our four basic criteria for FAIRness, which suggests a need to support these groups to enact basic steps to improve the resource's adherence to the FAIR principles. Applying a community-developed data capture or transfer standard for meta-level or participant-level data is the most resource-intensive and least commonly enacted of the four criteria. The role of data and metadata standards as essential elements for the consistent and meaningful reporting and sharing of information precedes the FAIR principles, and their patchy implementation and use is a known issue.^52^, ^53^ Key challenges for interoperable clinical or epidemiological participant-level data and metadata include: (1) fragmentation with gaps and duplications and a lack of intra standard interoperability, which limits their consistent use, especially between medical and research areas; (2) differences in the governance and terms of use, particularly between formal standard organisations and grass-root initiatives, which often limits contributions, extensions, and modifications; (3) a lack of funds to implement the standards for participant-level data, train users, curate data, and support the standards life cycle, which is necessary to deal with the evolving technologies and emerging data types; and (4) a lack of standards for study metadata. In this analysis, we were unable to directly measure the uptake of community-developed standards by data resources and had to collect information on resource adoption of standards with an online survey and web searches. This snapshot of the standards landscape, which will continue to evolve on FAIRsharing.org, should facilitate conversations about the wider adoption of common standards and the need for cross-standard interoperability.

### Cross-registry or cross-platform interoperability of participant-level data

The use of community-developed standards for participant-level data and study metadata is an important precondition for interoperable data. The use of different community-developed standards for participant-level data might be unavoidable and could be addressed retrospectively, by application of the Observational Medical Outcome Partnership Common Data Model (OMOP CDM).^54^ However, few platforms or registries applied community-developed standards for participant-level data, further restricting the interoperability of these data-sharing initiatives.

Comprehensive, machine-readable study and data-sharing resource metadata (discovery and descriptive metadata on data provenance, subject area, collection, contents, accessibility, etc) are the first step towards interoperability. Funders might consider extending ongoing efforts to develop guidelines for user-defined metadata,^55^ with a focus on clinical metadata, where, in contrast to omics and high-dimensional imaging data, key metadata are not defined at data capture. Interoperability of platform or registry metadata and the application of shared standards for participant-level data would represent important progress towards inter-platform or inter-registry interoperability.

### Ethical concerns and compliance with data protection laws

Ethical or governance-related concerns should be addressed. We reviewed several disparate frameworks for evaluating ethical concerns when sharing participant-level research and EMR data,^16^, ^17^, ^18^ including two that were specific to sharing data in response to a public health emergency.^14^, ^15^ Although there is general agreement that broad consent for future use should be sought when sharing de-identified EMR or research data,^1^ some groups argue that broad consent, and even informed consent, is not needed for sharing de-identified data.^56^, ^57^ Where broad consent for future use was not possible or sought, a waiver of consent could be granted for sharing participant-level data in keeping with the Council for International Organizations of Medical Sciences guidance.^58^ Most countries have legal frameworks for sharing participant-level data in the public health response to an emergency, such as the COVID-19 pandemic, irrespective of consent.^59^

Most registries and almost half of the platforms specified that they only collected de-identified data; most of these registries also indicated that they were exempt from ethical review because they were only sharing de-identified data. Maintaining data utility while preventing re-identification is a major challenge, especially in the COVID-19 response where participant-level linkages between data types (ie, pathogen and host omics data and clinical data) are important for detecting and responding to VOCs. Different definitions of what anonymised and pseudonymised data means further complicate cross-initiative discussions and approaches.^7^ Data sharing resources should consider establishing an independent ethics advisory committee, as distinct from a research ethics committee, that reflects community values and preferences for data sharing and that can evaluate key ethical issues. Interoperable governance, consistent definitions, and common approaches to shared ethical and legal issues would both conserve scarce resources and facilitate explicit connections between related data sharing investments.

### Equitable distribution of platforms

Multiple groups have highlighted the dangers of parachute research in the context of data sharing^60^, ^61^ and indicated that data sharing is perceived as widening existing disparities in access to funding and publication opportunities between researchers in high-income countries and LMICs.^62^ Platforms, in particular, represent long-term, major investments in infrastructure and specialised expertise, and the absence of data sharing platforms in LMICs represents a missed opportunity to support equitable, global data sharing for COVID-19 response.

### Community engagement and benefit sharing

Resources that collect, harmonise, and share data have to be responsive to competing needs from diverse stakeholders, including data generating groups, research participants and their source communities, funders, and end-users whether they are academic or commercial, the general public, or the Open Science Community. Community engagement is central to ethical data use and ensuring meaningful benefit sharing.^63^ When conducted properly, community engagement engenders trust, fosters understanding and ownership, and promotes the partnerships with communities that can support both data sharing and future research.^64^ In our Review, some of the most frequently reported forms of benefit sharing were data dashboards and acknowledgment of the data contributing groups or co-authorship on publications using their data. Benefit sharing could also be in the form of documentation of data sharing-facilitated knowledge translation that could empower governments, the medical community, or the general public to take early action during a pandemic.^65^ Fewer than a quarter of registries and platforms reported engaging communities or investing in research capacity building.

### Transparent governance

Data access models correspond to different political, ethical, administrative, regulatory, and legal contexts, resulting in different systems for the review and assessment of proposals to access the data. A common system to manage access involves the consideration of a data access application by a centralised DAC. DACs review and evaluate proposals to access data and are central to ensuring that community values and preferences are reflected in data sharing decision making and setting public health priorities for data reuse.^66^ Independent commissions (eg, DACs) rather than individual researchers should be responsible for ensuring fair and equitable data sharing that balances the interests of data providers (eg, publications), research participants or patients, and the open science and public health communities.^67^ Of the 18 platforms and 24 registries that are or will share participant-level data, six (33%) platforms and 17 (71%) registries included a DAC.

Several reviews explore best practice for DACs,^13^, ^17^, ^68^, ^69^ which include, at a minimum, community representation, transparency and consistency regarding the process, criteria, and decisions regarding data requests and specific steps to avoid conflicts of interest between DAC members and dataset applicants. Further work is needed to define best practice for data governance with a focus on interoperable governance of data sharing efforts when responding to a Public Health Emergency of International Concern. In public health emergencies, software approaches to shielded data access (eg, DataSHIELD),^70^, ^71^ which allow for analysis without end users moving or seeing the data, might be a way to address ethical and legal concerns and ensuring timely data access for informed public health response.

### Legal barriers to data sharing

Concerns about recent data protection laws, including the General Data Protection Regulation (GDPR), might be correlated to siloed data and governance efforts, as when a platform deputises individual institutions to manage data access rather than pooling responsibilities arising from data protection law, including establishing a centralised DAC to avoid distributed controllership. A lack of clarity in terminology^7^ has contributed to inconsistent interpretations and applications of data protection laws within and beyond Europe, which further hinders the interoperability of governance structures and initiatives that share interconnected data types. These fears have persisted despite provisions to support data sharing in response to public health emergencies,^72^ including article 9(2)i of the GDPR, which allows for the processing of sensitive personal data for reasons of public interest in the area of public health, including protection against serious cross-border threats to health, and article 49(1)d of the GDPR, which provides an exemption for international data transfers if necessary for important reasons of public interest, which in practice includes the public health response to infectious diseases.

Many countries do not have national legal frameworks related to the cross-border transmission and transfer and sharing of participant-level health-related data. As for the GDPR, its scope of application is broad and often results in the requirement for research entities in countries outside of Europe to comply with GDPR when interacting with EU-based institutions when submitting, accessing, or receiving participant-level health data. The application of GDPR to the data processing activities of international organisations actively contributing to health research is contested. However, if EU-based organisations share data with international organisations, they should check the level of data protection within these organisations, which should be equivalent to GDPR-level protection. Thus, besides the scope of application, transfer rules also quickly extend the reach of GDPR, making it, on a practical level, the default data protection legislation. Additionally, collision rules are unclear when legal frameworks that prescribe data governance interact across national boundaries that leads to confusion regarding which rule to apply to the same data or a jointly conducted research activity that might further hinder data sharing.

### Quantifying data resource utility

The public health imperative to share data to improve COVID-19 prevention and response has led to a growth of data-sharing platforms and registries. There is a real need to understand the return on investment for these data-sharing initiatives and to inform strategies to maximise the utility and sustainability of existing initiatives. Although there have been several case studies that seek to show the utility of data sharing platforms, efforts to describe the public health-related benefits of sharing harmonised participant-level health-related data have been largely qualitative. Future research could identify markers for contributions to and use of data sharing platforms and how the harmonisation and dissemination of data facilitate research translation, build scientific networks, and lead to new fields of inquiry. In addition to understanding the utility of data-sharing initiatives, clear metrics and quantitative approaches to assessing the downstream benefits and harms of data sharing could facilitate an exploration of ethical issues, such as whether data generated by researchers in LMICs benefit their communities and whether data contributors receive some measurable advantage in terms of novel funding applications, publications, collaborations, or research directions from data sharing and producing the metadata needed to appropriately interpret the shared data.

### Identifying and supporting successful investments

Platforms, and to a lesser extent, registries, require considerable investment of money and time. For example, the IDDO platform began with the Worldwide Antimalarial Resistance Network in 2004, and an initial investment of over US$20 million.^73^ Investments in developing the governance and infrastructure for platforms that pre-dated the COVID-19 pandemic helped the platforms transition rapidly to COVID-19 data collection. Although established platforms, such as IDDO, had shared data on close to 500 000 participants,^74^ many COVID-19 platforms created during the pandemic were not yet sharing data in July 2021, when the platforms and registries overview dataset was finalised for the initial journal submission. Understanding which data-sharing resources are successful in collecting and sharing data is as important as understanding how resources map to the FAIR principles and best practices for ethical considerations related to international data sharing. We documented several metrics resources used for evaluating their resource's utility, including the number of datasets, participants, genome sequences, and users. Most platforms were supported by government funding, and NGOs or professional organisations were the most common sources of funding for registries. Funders should consider implementing standardised, externally verifiable, and metadata-driven measures to evaluate the success of data reuse resources in attracting data submissions and generating data reuse requests when supporting established resources or investing in new platforms and registries.

CTS is perhaps the most recognised framework for understanding how data sharing resources adhere to best practice. Most of the resources that we identified would not have been eligible to apply for CTS because they did not state their commitment to data preservation and providing access to data. Of those resources that were eligible to apply for CTS, only one resource, established in 2004,^32^ had received certification. CTS is used more widely in social sciences and for astronomy, environmental, geospatial data, or is used by institutional repositories that take a data lake approach rather than in biomedical research. Although FAIRsharing.org and R3Data provide basic discovery metadata related to the limited number of biomedical resources that register with their sites, more work is needed to help researchers and funders identify trusted registries and platforms. The low uptake of the CTS certification in the biomedical data resource space might be related to the high cost and administrative burden associated with certification, the lack of awareness of CTS, or the absence of research and funder focus on metrics and frameworks for evaluating and comparing repositories. Given that many of the registries that we identified in our search were initiated and managed by professional organisations (eg, the American Heart Association), facilitated discussions within and across professional organisations to identify key priorities for ensuring registries reach their intended audiences might be the best way to identify trusted data reuse resources to allocated limited funding for data preservation and sharing.

### Coordinated data sharing for COVID-19 response

Collaboration between data-sharing efforts, focusing on the technical interoperability of related resources on the basis of the semantic interoperability of participant-level data and metadata from shared use of community-developed standards, is perhaps the most crucial area for investment. The aggregation of standardised data across interoperable platforms or registries would help move towards the types of shared global analyses that could meaningfully inform the response to a global pandemic. Applying the same or interoperable standards for related study-level and participant-level data is a necessary, but insufficient condition for inter-resource interoperability. In a few instances, connected platforms mean that data uploaded to one platform are reflected in another platform (eg, SARS-CoV-2 omics data uploaded to the European Molecular Biology Laboratory–European Bioinformatics Institute COVID-19 Data Portal or the National Center for Biotechnology Information is included in International Nucleotide Sequence Database Collection), which enhances data findability and reuse. Large initiatives have emerged to connect platforms and registries within countries and regions, including the Health Data Research UK Innovation Gateway and the European COVID-19 Data Portal. Several initiatives exist to catalogue both COVID-19 data-sharing initiatives and datasets (eg, FAIRsharing and the covid19dataindex). Coordination of COVID-19 clinical data-sharing initiatives should include: the identification of several core common data models, which can be meaningfully applied to research and EMR data; best practice for governance and addressing ethical and legal concerns, which can form the basis of an interoperable governance structure and common approach, where possible, to shared ethical and legal issues; and improved technical approaches for querying related data shared on disparate platforms or registries, including shielded approaches whereby participant-level data can be analysed without being downloaded from the platform. Interoperability-focused initiatives that improve access to FAIR clinical and human and pathogen omics and high-dimensional imaging data should be prioritised to facilitate the global response to VOCs.

Public health emergencies remind the public health and scientific communities of the urgent need to address unresolved barriers to sharing data in the context of infectious disease outbreaks. In contrast to the Zika and Ebola virus outbreaks, COVID-19 has ushered in a new era whereby researchers and funders need to shift their focus from supporting data sharing to promoting coordination between data sharing activities. Access to health data was on the policy agenda before the COVID-19 pandemic.^75^ The G8 established an Open Data Charter in 2013, to promote, in part, the availability of health-related data.^76^ Interoperability and access to EMR data for improved care at the patient and population level and to enable patients to access their own health data had also been supported by large investments and health ecosystem-related legislation in the USA,^77^, ^78^ Europe,^43^, ^79^, ^80^ Asia,^81^ Latin America,^82^ and Africa.^83^, ^84^ Despite these commitments, a subsequent G7 report highlighted the gaps between what was needed and what was implemented for rapid access to high-quality data at the beginning of the COVID-19 pandemic.^85^

Building on these previous investments and legislation, the data sharing community, including funders, researchers, hospital networks, and public health authorities, need to move from a reactionary, fragmented response to a coordinated, synergistic approach across platforms and registries that facilitate access to harmonised, participant-level COVID-19 data. Ensuring that data sharing resources are as FAIR as possible and best practices for resource governance, transparency, community engagement, applicable legal frameworks, and recommended ethical (eg, protection of research subjects and ERC review) and equitable practice (eg, benefit sharing and community engagement) continue to be key concerns. In particular, interoperability within and between types (eg, clinical, laboratory, and omics) and sources (eg, EMR and research studies) of data should be a top priority for current and future epidemics. Cloud-based platforms for data sharing represent a tremendous investment of financial resources and expertise. Clearly elaborated criteria for identifying successful platforms that apply best practice for governance and addressing ethical concerns, including benefit sharing, while meaningfully engaging the community can help funders focus investment by supporting good practice. Although some duplication of effort should be expected, the ecosystem of 44 registries and 20 platforms for collecting participant-level COVID-19 data that are not interoperable represents a lost opportunity and wasted resources. Given clear criteria for assessing resources, funders, data-generating groups, and the open science community can focus on a smaller number of well supported platforms and registries. Identifying the key political, ethical, administrative, regulatory, or legal motivations for the creation of disparate, non-interoperable platforms for different diseases and data types is important for preventing continued investment in siloed data-sharing efforts. Data-sharing platforms generally have large budgets because of the high cost of platform development and maintenance, retrospective data harmonisation, and the governance of data sharing. All data sharing platforms, except for the China National GeneBank DataBase, were based in high-income countries, which raises questions of equity in the distribution of resources, concerns about the appropriate representation of the values and preferences of research teams and participants based in LMICs, and in opportunities to build expertise in data curation and sharing. Data sharing is clearly on the policy agenda. We now need to move from fragmented, overlapping, and competing data-sharing efforts to a coordinated nexus of interconnected and longitudinal participant-level data. Given the formidable barriers for such a cross-regional, cross-discipline initiative, we should start work now to be ready for the next global pandemic.

### Search strategy and selection criteria

We conducted a monthly search of Google and Google Scholar between May, 2020, and June, 2021, for COVID-19 and for data sharing resources to identify relevant platforms and registries that collect, harmonise, and share COVID-19-related participant-level clinical, human or pathogen omics, and high-dimensional imaging data. The search terms were: “coronavirus OR COVID-19 OR severe acute respiratory syndrome OR coronavirus-2019 OR nCoV OR 2019nCoV OR 2019-novel CoV OR corona vir* OR coronavir* OR neocorona vir* OR neocoronavir* OR COVID OR COVID19 OR nCov 2019 OR nCov 19 OR SARS-CoV-2 OR SARS-CoV2 OR SARSCoV2 OR SARSCoV-2 SARS coronavirus 2 OR SARS-like coronavirus OR Severe Acute Respiratory Syndrome Coronavirus-2” AND “database OR databases OR repository OR repositories OR registry OR registries OR platform OR platforms”. To account for English-language bias in the search strategy, we contacted investigators that work on COVID-19-related data sharing in Asia, Africa, and Latin America and applied natural language processing (NLP) to the COVID-19 Open Research Dataset in March, 2021, to identify additional data sharing resources. We applied rule-based syntactic matching with the spaCy NLP software package in Python. NLP code is available on GitHub and the NLP approach is described in the appendix (p 3). We extracted data related to resources that: (1) collected and harmonised (prospectively or retrospectively) participant-level COVID-19-related health data; (2) included data from more than one hospital, hospital system, or study; and (3) had a website. We did not include data lakes, data verses, catalogues of datasets, or other data resources that do not harmonise participant-level data. We also excluded multicentre cohort studies whereby sites were set at the beginning of the study and the inclusion of new sites was not allowed. Because of the ongoing and iterative nature of the search and the dependence on grey literature sources, we did not produce a PRISMA flow diagram.

### Data sharing

The dataset describing the 64 platforms and registries that collect, harmonise, and sometimes share COVID-19-related participant-level clinical, omics, and imaging data is available for comment on Zenodo. Metadata for these resources are available on FAIRsharing via a dedicated collection.

## Declaration of interests

LM received grants from Tropical Disease Research via WHO (P20-00007), the ReCoDID project (which is funded by the EU Horizon 2020 Research and Innovation Programme; 825746), and the Canadian Institutes of Health Research Institute of Genetics (01886-000). S-AS received a grant from FAIRsharing, which is funded by the Wellcome Trust (212930/Z/18/Z). The funder had no role in study design, data collection, data analysis, data interpretation, writing of the manuscript, or the decision to publish. All other authors declare no competing interests.
