# Supplementary material for: FAIR, ethical, and coordinated data sharing for COVID-19 response: a scoping review and cross-sectional survey of COVID-19 data sharing platforms and registries
Source: Lancet Digit Health. 2023 Sep 27;5(10):e712–36. doi: 10.1016/S2589-7500(23)00129-2 (PMC10552001; doi:10.1016/S2589-7500(23)00129-2)
Supplement: Supplementary appendix [file mmc1.pdf]

THE LANCET
Digital Health

Supplementary appendix

This appendix formed part of the original submission and has been peer reviewed. We post it as supplied by the authors.

Supplement to: Maxwell L, Shreedhar P, Dauga D, et al. FAIR, ethical, and coordinated data sharing for COVID-19 response: a scoping review and cross-sectional survey of COVID-19 data sharing platforms and registries. *Lancet Digit Health* 2023; **5:** e712–36.

FAIR, ethical, and coordinated data sharing for COVID-19 response: a scoping review and cross-sectional survey of COVID-19 data sharing platforms and registries

Supplementary Material

Table of Contents

Research in context 1

Supplementary Table 1. Working definitions for resources for sharing participant-level data. 2

Supplementary Note 1. Natural Language Processing Strategy & Source Code 3

Supplementary Note 2. Quantitative evaluation of the adherence of registries with clinical participant-level COVID-19 data to the FAIR Principles 3

Supplementary Table 2. Application of FAIRshake[11] algorithm to registries for sharing COVID-19-related participant-level clinical data 7

Preferred Reporting Items for Systematic reviews and Meta-Analyses extension for Scoping Reviews (PRISMA-ScR) Checklist 10

Supplementary Table 3. Potential data sharing resources identified through application of NLP to CORD-19 database 12

Supplementary Figure 1. Registry and platform-specific linkages between data types 21

Supplementary Figure 2. Summary distribution of platforms and registries for sharing participant-level COVID-19-related health data* 22

Supplementary Figure 3. Distribution of platforms and registries for sharing participant-level COVID-19-related health data 23

Supplementary Table 4. Summary of principles from health data sharing frameworks 24

Supplementary Table 5. Platforms and registries associated with Figure 1 27

Supplementary Figure 4. Standards-based and technical interoperability between all of the COVID-19 data sharing resources 32

Supplementary Figure 5. Standards-based and technical interoperability between the COVID-19 registries 33

Supplementary Figure 6. Standards-based and technical interoperability between the COVID-19 platforms 34

References 35

Research in context

Evidence before the study

We identified a descriptive review of cancer-related registries of COVID-19 patient data. However, to the best of our knowledge, this paper is the first comprehensive review of how efforts to improve the availability of harmonized, participant-level COVID-19 data correspond to the findable, accessible, interoperable, reusable (FAIR) principles and other frameworks for data sharing during and outside of epidemics.

Added value of this study

Understanding how participant-level data are shared can help funders and researchers identify gaps and redundancies to improve global collaboration in the research response to COVID-19. In this manuscript, we present an in-depth overview of the ever-expanding universe of COVID-19-related platforms and registries for sharing participant-level clinical, OMICs, and imaging data and review how these initiatives map to best practices for ethical, equitable, and effective data sharing as well as the FAIR principles for data resources. In large part, platforms and registries for improving the availability of harmonized clinical or epidemiological, participant-level COVID-19 data had not adopted community developed standards for participant-level data and were often siloed by data type, comorbidity, body system, and population type.

Implications of all the available evidence

To better respond to the ongoing pandemic, we need to move from fragmented, overlapping and competing data sharing efforts to a coordinated nexus of interconnected, longitudinal, participant-level data.

Supplementary Table 1. Working definitions for resources for sharing participant-level data

Term	Definition	Approach to harmonization	Data types
Platform[1]	Combines big data tools and infrastructure. Major investment to continuously store, manage, mine big data sets (e.g. OMICs, imaging data).	Retrospective or prospective	May be limited to 1 data type or include various prespecified data types
Registry[2]	Collection of data stored in an assigned location. Low level of investment needed. Data generally entered or uploaded using the same case report form/data dictionary and the focus is on a particular disease, condition, or exposure.	Prospective	Generally limited to 1 specific data type
Dataverse[3]	Open source web application to share, preserve, cite, and explore research data of various types and with varying objectives.	Data is in its original form and not harmonized	Any
Datahub[4]	Data store that is an integration point for multiple datasets with different structures. Data are moved and stored together, however access permissions vary by data contributor.	Generally involves harmonization of data	Any
Data lake[4]	Central repository or pool of raw and untransformed data of any data type for an undefined purpose and requires other add-on tools to search or operationalize the data. Requires a low-level of investment.	Data is in its original form and not harmonized	Any
Data warehouse[5]	Data management tool that contains structured, filtered data that has already been processed and refined for a specific purpose allowing end users to perform further analytics.	No harmonization	Any
Data federation[6]	Technology wherein the data stored in different data sources are made accessible as one integrated virtual database and can be queried, transformed and accessed by data consumers. Data federation is a subset of data virtualization.	Data federation involves transformation, cleansing, and at times, the enrichment of data	Any
Data virtualization[4]	Data virtualization evolved from data federation with additional features and functionalities. According to different software developers, data virtualization has several capabilities beyond data federation including advanced security, query processing, and data transformation features.	Same as data federation	Any
Data catalogue[7]	Website with linkages to available datasets or platforms.	No harmonization	Does not host data

Supplementary Note 1. Natural Language Processing Strategy & Source Code

We applied natural language processing (NLP) to the Covid-19 Open Research (CORD-19) Dataset[8] to identify additional COVID-19-related data sharing platforms and repositories. NLP was a useful approach to dealing with the CORD-19 resources in English as well as different languages because processing this data through automation is difficult to do without an understanding of the way humans speak and write naturally. We used two main methods to match potential titles containing COVID-19 related data sharing platforms. Firstly, we looked at proper nouns, serving the roles of named entities and acronyms, which take the role of the root of the sentence. Additionally, we matched appositional modifiers to the target search terms to pick up any missed items that the initial algorithm did not pick up, due to the format the title was written in its respective language.

The initial NLP was conducted in a Jupyter Notebook environment using R. For the initial NLP approach, we singled out titles in the CORD-19 database with desired relatability to the publication using the keywords: registry, registries, database, databases, platform, platforms, repository, repositories, IPD-MA, individual participant data meta-analysis, and data dashboard. The original NLP R source code can be found at the following link: https://github.com/matiasbross/NLPCode.

We later updated the NLP approach using the open-sourced Python NLP library - SpaCy.[9,10]
We singled out titles in the CORD-19 database with desired relatability to the publication using the keywords: registry, registries, database, databases, platform, platforms, repository, repositories, IPD-MA, individual participant data meta-analysis, and data dashboard.
The updated NLP Python source code can be found at the following link:
https://github.com/AdmiralVanko/Cord19-Scraper.

Supplementary Note 2. Quantitative evaluation of the adherence of registries with clinical participant-level COVID-19 data to the FAIR Principles

We limited the quantitative evaluation to registries for participant-level clinical data because we could not apply the same metrics for resources that shared different data types. For example, participant-level clinical registries have restricted access due to the sensitive nature of the data whereas databases for sharing pathogen OMICs data are open access. While discipline-specific FAIR criteria should be developed using a diverse panel of experts and stakeholders, we applied indicators used by the FAIRshake tool[11] algorithm to better align the tool's evaluation with the specific concerns that we thought would be most important to end users of registries of clinical data. Only one of the registries that collect and harmonize COVID-19 participant-level clinical data had been assigned a DOI prior to our review of the registries. Eighteen of the registries were assigned a DOI by FAIRsharing as part of our evaluation. Seventeen of the registries that we contacted to assign a DOI did not respond to these inquiries, and we could not quantify their FAIRness.

Below, we review the criteria used to create a preliminary rubric for evaluating registries' adherence to the FAIR principles. These draft criteria will be presented to the Research Data Alliance, an international network of individuals and groups working to improve FAIR data. Blue text indicates metrics from our research team's dataset. Green text is used for metrics from the FAIR Data Maturity Model Specification and Guidelines 2020.[12] Text with a strikethrough indicates text that was removed from the corresponding indicator in our dataset.
Preliminary criteria for the application of the FAIR assessment rubric
Findable

- PID (unique & persistent identifier) for the data (RDA-F1-01D / RDA-F1-02D)

Does the repository provide PIDs for the datasets therein?
Value: Values will be the same for all the COVID-19 resources we assessed as it isn't clear (in a machine-actionable manner) what kind of PID they use, as we don't have data access. This means all the registries will fail this indicator.

- Annotation with metadata (RDA-F2-01M)

This is the only thing that could differ between resources: the quality / quantity of metadata annotation.
We can consider 3 levels:
i) nothing -> fail
ii) minimum (contact, description, to be defined) -> medium
iii) rich (? to be defined)
This could also be the sum of the criteria filled out in the WHO survey.
Value: Consider the metric as a success for every registry, as this was a criterion to enter them in FAIRsharing (a minimum set of metadata must be required to be inserted into FAIRsharing).

- PID (unique & persistent identifier) for the metadata (RDA-F1-01M / RDA-F1-02M)

Does the metadata from the repository are assigned a unique & persistent identifier?
Value: Values will be the same for all the COVID-19 resources because they have a PID for metadata **in FAIRsharing**.

- Link between PID_data & PID_metadata (RDA-F3-01M)

Does the metadata include the unique & persistent identifier of the data?
Value: this will always give a failure (PID_data: FAIL ; PID_metadata: FAIL) .

- Findable on search engines (RDA-F4-01M)

Are the registries findable on search engines? (we can check if they are marked up with Schema.org
Did not assess whether the registers were present on portals or institutional websites.
Value: every registry gave a success except "European Renal Association COVID-19 Database" (when searching on Google, can't find https://www.eracoda.org/ link, but I can access thanks to FAIRsharing link or others websites that redirect to the link).

Accessible

- Standard protocol and secured standard protocol (https, ftps) (RDA-A1-04M / RDA-A1.1-01M)

Is the metadata accessible via standard protocols such as HTTPS and FTPS?
Value: Values are the same for all the registries, as they can be accessible by https website.

- Authentication secure (RDA-A1.2-01D)

Is sensitive data accessible by secure authentication?
Value: Consider REDCap secure (=success). For registries we don't know if there is an authentication (REDCap is not used), assigned a "Not Clear" value for these cases.

- Metadata accessibility on the long term (RDA-A2-01M)

Will the metadata be accessible in the long term even if the resource disappears?
Value: Values will be the same for all the COVID-19 resources because all the resources have a PID for metadata on FAIRsharing.

- Contact information (no correspondence with RDA)

Is there any contact information available on the website (not sure that we should make a distinction between a "registry contact" and a PI contact: a registry contact is better for sustainability but there is a chance that these rapidly emerging resources will disappear just as quickly and, in this case, a PI contact is better).
"Registry email"
"PI email(s)"

“Registry contact email(s)”

Value: Marked as successful only if one of these criteria is met.

- Contact information valid (no correspondence with RDA)

In the context of this type of repository, it is important that the contact responds. The WHO team sent a survey to the contact and they received or not an answer. I think we can only consider “responded to survey.”
“Responded to survey with detailed questions about data types, sharing, and governance”
“Notes from investigator on how to access data”
Value: Marked as successful if “Responded to survey with detailed questions about data types, sharing, and governance” is met.

- Data access (RDA-A1-01M)

Is there a clear description of the access to the data?
“Link to description of how to access data”
“Link to clearly specified governance mechanism for reviewing data access requests”
“Link to clearly specified criteria for reviewing data access requests”
“Criteria for reviewing data access requests (from REDCap)”
“Who controls access to the data”
Value: Ignored “Criteria for reviewing data access requests (from REDCap).” Averaged the other 4 criteria (green: 1 ; red: 0 ; yellow: 0,5).
Removed the “who controls access to the data,” it doesn’t bring anything.

- Data sharing (no correspondence with RDA)

Is the data shared? As raw data is not directly accessible, here we can assess if some summary / reports / data dashboard / scientific articles are available.
“Data sharing status”
“Investigator explanation for why data won't be shared (write N/A if data will be shared)”
“Is there a data dashboard / articles / reports available?”
Value: Ignored “Investigator explanation for why data won't be shared (write N/A if data will be shared).” Marked as successful only if one of the two criteria is met.
Interoperable

- Use of a controlled vocabulary (RDA-I1-01D)

Does the data use a knowledge representation expressed in a standardised format? It can be assumed here that the use of forms to insert patient data allows the use of a controlled vocabulary.
“Link to COVID-19 CRF or data dictionary”
Value: Success only if one of the two criteria is met.

- Use of a FAIR controlled vocabulary (RDA-I2-01M / RDA-I2-01D)

Does the data use a knowledge representation expressed in a FAIR standardised format? We can remove OMICS standards and Imaging data standards because not appropriate for these registries.
“What formal standards does the platform apply for human OMICs data access?”
“Connection between CRF and existing standards (e.g., ICD 9-11, CDASH, SNOMED, LOINC)”
“Uses ISARIC/WHO CRF (case report form)?”
“Clinical-epidemiological standards used by registry”
“OMICs data shardards used by registry”
“Imaging data standards used by registry”
Value: no known standard could be identified in the registries (except for the Extracorporeal Life Support Organization Registry which uses a Clinical-epidemiological standard)
Removed OMICS and imaging standards as we only look at registries.

Removed the connexion between CRF and existing standard (removed from WHO spreadsheet + doublon with the use of clinical-epidemiological standards used by the registry).

- Data contextualisation (related resources) (RDA-I3-01M)

Are there links to platforms in the same field to contextualise the register? Are there links to clinical trials on ClinicalTrials.gov?
"Links to related platforms"
Value: success if yes, failure if no.
Reusable

- Licence (RDA-R1.1-01M)

Is there a clear and accessible licence for re-use?
"Data usage license"
Value: success if yes, failure if no. Yes if found a link "terms of use," "terms of service," "copyright notice" on the corresponding website

- Source of data (no correspondence with RDA)

Does metadata include provenance information?
"Who can enter data? (anyone, registered users of the platform, the platform hosts)"
"How is data entered? (can data be uploaded? Is this through a REDCap data entry platform, etc?)"
Value: I averaged the 2 criteria.

- Use of community standard (RDA-R1.3-01M, RDA-R1.3-01D / RDA-R1.3-02M / RDA-R1.3-02D)

Does data and metadata comply with a community standard? Is data and metadata expressed in compliance with a machine-understandable community standard?
These are the results of: "Use of a FAIR controlled vocabulary." Almost all registries missed meeting this criterion.
Value: Took the same results as the "Use of a FAIR controlled vocabulary" criteria (meaning failure for most of the registries). I just improved the score of the "except for the Extracorporeal Life Support Organization Registry" and "Discovery VIRUS COVID-19," as one standard is not sufficient to meet this criterion.

Supplementary Table 2. Application of FAIRshake[11] algorithm to registries for sharing COVID-19-related participant-level clinical data

	ASCO Registry	COVID-19 CVD Registry	COVID-HEP Registry	COVID-19 Dermatology registry	Discovery VIRUS COVID-19	Extracorporeal Life Support Organization Registry	Pregnancy Coronavirus Outcomes Registry	COVID-19 Global Rheumatology Alliance Registry	SECURE-Liver Registry	ASH Research Collaborative COVID-19 Registry for Hematologic Malignancy	Coronavirus and MS Reporting Database	SECURE-Celiac	SECURE-Psoriasis	SECURE-Sickle Cell Disease	SECURE-Atopic Dermatitis / SECURE-Alopecia	MS Global Data-Sharing Initiative	The European Renal Association COVID-19 Database
**FINDABLE**																	
PID (unique & persistent identifier) for the data	Red	Red	Red	Red	Red	Red	Red	Red	Red	Red	Red	Red	Red	Red	Red	Red	Red
Annotation with metadata	Green	Green	Green	Green	Green	Green	Green	Green	Green	Green	Green	Green	Green	Green	Green	Green	Green
PID (unique & persistent identifier) for the metadata	Green	Green	Green	Green	Green	Green	Green	Green	Green	Green	Green	Green	Green	Green	Green	Green	Green
Link between PID_data & PID_metadata	Red	Red	Red	Red	Red	Red	Red	Red	Red	Red	Red	Red	Red	Red	Red	Red	Red
Is the repository findable on search engine?	Green	Green	Green	Green	Green	Green	Green	Green	Green	Green	Green	Green	Green	Green	Green	Green	Yellow
**ACCESSIBLE**																	
Standard protocol (http, ftp, smtp)	Green	Green	Green	Green	Green	Green	Green	Green	Green	Green	Green	Green	Green	Green	Green	Green	Green
Secured standard protocol (https, ftps)	Green	Green	Green	Green	Green	Green	Green	Green	Green	Green	Green	Green	Green	Green	Green	Green	Green
Authentication secure (RedCap is considered as secure)	Green	Yellow	Green	Green	Yellow	Yellow	Green	Green	Yellow	Yellow	Green	Yellow	Green	Green	Yellow	Yellow	Green
Metadata accessibility long term	Green	Green	Green	Green	Green	Green	Green	Green	Green	Green	Green	Green	Green	Green	Green	Green	Green
Contact information (registry or PI email)	Green	Green	Green	Green	Green	Green	Green	Green	Green	Green	Green	Green	Green	Green	Green	Green	Green
Contact information valid	Red	Green	Red	Red	Red	Red	Red	Green	Green	Green	Red	Green	Red	Red	Green	Red	Red
DATA ACCESS: Link to description of how to access data	Green	Green	Red	Green	Green	Green	Red	Green	Red	Red	Red	Red	Red	Red	Red	Red	Green

	ASCO Registry	COVID-19 CVD Registry	COVID-HEP Registry	COVID-19 Dermatology registry	Discovery VIRUS COVID-19	Extracorporeal Life Support Organization Registry	Pregnancy Coronavirus Outcomes Registry	COVID-19 Global Rheumatology Alliance Registry	SECURE-Liver Registry	ASH Research Collaborative COVID-19 Registry for Hematologic Malignancy	Coronavirus and MS Reporting Database	SECURE-Celiac	SECURE-Psoriasis	SECURE-Sickle Cell Disease	SECURE-Atopic Dermatitis / SECURE-Alopecia	MS Global Data-Sharing Initiative	The European Renal Association COVID-19 Database
DATA ACCESS: Link to clearly specified governance mechanism for reviewing data access requests																	
DATA ACCESS: Link to clearly specified criteria for reviewing data access requests																	
DATA SHARING: Is/will the data be shared																	
DATA SHARING: Is there a data dashboard / articles / reports available?																	
**INTEROPERABLE**																	
USE OF A CONTROLLED VOCABULARY: Link to COVID-19 CRF or data dictionary																	
USE OF A CONTROLLED VOCABULARY: Uses ISARIC/WHO CRF?																	
USE OF A CONTROLLED VOCABULARY: Clinical-epidemiological standards used by registry																	

	ASCO Registry	COVID-19 CVD Registry	COVID-HEP Registry	COVID-19 Dermatology registry	Discovery VIRUS COVID-19	Extracorporeal Life Support Organization Registry	Pregnancy Coronavirus Outcomes Registry	COVID-19 Global Rheumatology Alliance Registry	SECURE-Liver Registry	ASH Research Collaborative COVID-19 Registry for Hematologic Malignancy	Coronavirus and MS Reporting Database	SECURE-Celiac	SECURE-Psoriasis	SECURE-Sickle Cell Disease	SECURE-Atopic Dermatitis / SECURE-Alopecia	MS Global Data-Sharing Initiative	The European Renal Association COVID-19 Database
Data contextualisation (related resources): Links to related platforms	Green	Red	Green	Green	Red	Red	Green	Red	Green	Green	Red	Green	Green	Red	Green	Red	Red
**REUSABLE**																	
License	Green	Green	Red	Red	Green	Green	Green	Red	Red	Green	Red	Red	Red	Red	Red	Green	Red
SOURCE OF DATA: Information about who can enter data (anyone, registered users of the platform, the platform hosts)	Green	Green	Green	Green	Green	Green	Green	Green	Green	Green	Green	Green	Green	Green	Green	Green	Green
SOURCE OF DATA: Information about how is data entered (can data be uploaded? Is this through a REDCap data entry platform, etc?)	Green	Green	Green	Green	Green	Green	Green	Green	Green	Green	Green	Green	Green	Green	Green	Green	Green
Use of community standard	Red	Red	Red	Red	Yellow	Yellow	Red	Red	Red	Red	Red	Red	Red	Red	Red	Red	Red

Green: the FAIR criteria are met; Red: the FAIR criteria are not met; Yellow: insufficient information.
ASCO=American Society of Clinical Oncology. ASH=American Society for Hematology. CRF=case report form. CVD=cardiovascular disease. ftps=file transfer protocol secure. https=hypertext transfer protocol secure. ISARIC=International Severe Acute Respiratory and Emerging Infection Consortium. MS=multiple sclerosis. PI=principal investigator. PID=persistent identifier. SECURE=Surveillance Epidemiology of Coronavirus Under Research Exclusion. smtp=simple mail transfer protocol. WHO=World Health Organization.

Preferred Reporting Items for Systematic reviews and Meta-Analyses extension for Scoping Reviews (PRISMA-ScR) Checklist

SECTION	ITEM	PRISMA-ScR CHECKLIST ITEM	REPORTED ON PAGE #
TITLE			
Title	1	Identify the report as a scoping review.	1–2
ABSTRACT			
Structured summary	2	Provide a structured summary that includes (as applicable): background, objectives, eligibility criteria, sources of evidence, charting methods, results, and conclusions that relate to the review questions and objectives.	1
INTRODUCTION			
Rationale	3	Describe the rationale for the review in the context of what is already known. Explain why the review questions/objectives lend themselves to a scoping review approach.	1–2
Objectives	4	Provide an explicit statement of the questions and objectives being addressed with reference to their key elements (e.g., population or participants, concepts, and context) or other relevant key elements used to conceptualize the review questions and/or objectives.	2
METHODS			
Protocol and registration	5	Indicate whether a review protocol exists; state if and where it can be accessed (e.g., a Web address); and if available, provide registration information, including the registration number.	2
Eligibility criteria	6	Specify characteristics of the sources of evidence used as eligibility criteria (e.g., years considered, language, and publication status), and provide a rationale.	2,22
Information sources*	7	Describe all information sources in the search (e.g., databases with dates of coverage and contact with authors to identify additional sources), as well as the date the most recent search was executed.	2,22
Search	8	Present the full electronic search strategy for at least 1 database, including any limits used, such that it could be repeated.	22
Selection of sources of evidence†	9	State the process for selecting sources of evidence (i.e., screening and eligibility) included in the scoping review.	2,22
Data charting process‡	10	Describe the methods of charting data from the included sources of evidence (e.g., calibrated forms or forms that have been tested by the team before their use, and whether data charting was done independently or in duplicate) and any processes for obtaining and confirming data from investigators.	2,12
Data items	11	List and define all variables for which data were sought and any assumptions and simplifications made.	2,12
Critical appraisal of individual sources of evidence§	12	If done, provide a rationale for conducting a critical appraisal of included sources of evidence; describe the methods used and how this information was used in any data synthesis (if appropriate).	
Synthesis of results	13	Describe the methods of handling and summarizing the data that were charted.	2,12
RESULTS			
Selection of sources of evidence	14	Give numbers of sources of evidence screened, assessed for eligibility, and included in the review, with reasons for exclusions at each stage, ideally using a flow diagram.	6

SECTION	ITEM	PRISMA-ScR CHECKLIST ITEM	REPORTED ON PAGE #
Characteristics of sources of evidence	15	For each source of evidence, present characteristics for which data were charted and provide the citations.	Click here to enter text.
Critical appraisal within sources of evidence	16	If done, present data on critical appraisal of included sources of evidence (see item 12).	Click here to enter text.
Results of individual sources of evidence	17	For each included source of evidence, present the relevant data that were charted that relate to the review questions and objectives.	Table 1 (3-11) also available in a dataset uploaded to Zenodo)
Synthesis of results	18	Summarize and/or present the charting results as they relate to the review questions and objectives.	12-16
DISCUSSION			
Summary of evidence	19	Summarize the main results (including an overview of concepts, themes, and types of evidence available), link to the review questions and objectives, and consider the relevance to key groups.	16-23
Limitations	20	Discuss the limitations of the scoping review process.	16
Conclusions	21	Provide a general interpretation of the results with respect to the review questions and objectives, as well as potential implications and/or next steps.	16-23
FUNDING			
Funding	22	Describe sources of funding for the included sources of evidence, as well as sources of funding for the scoping review. Describe the role of the funders of the scoping review.	23

JBI = Joanna Briggs Institute; PRISMA-ScR = Preferred Reporting Items for Systematic reviews and Meta-Analyses extension for Scoping Reviews.

* Where *sources of evidence* (see second footnote) are compiled from, such as bibliographic databases, social media platforms, and Web sites.

† A more inclusive/heterogeneous term used to account for the different types of evidence or data sources (e.g., quantitative and/or qualitative research, expert opinion, and policy documents) that may be eligible in a scoping review as opposed to only studies. This is not to be confused with *information sources* (see first footnote).

‡ The frameworks by Arksey and O'Malley (6) and Levac and colleagues (7) and the JBI guidance (4, 5) refer to the process of data extraction in a scoping review as data charting.

§ The process of systematically examining research evidence to assess its validity, results, and relevance before using it to inform a decision. This term is used for items 12 and 19 instead of "risk of bias" (which is more applicable to systematic reviews of interventions) to include and acknowledge the various sources of evidence that may be used in a scoping review (e.g., quantitative and/or qualitative research, expert opinion, and policy document).

Supplementary Table 3. Potential data sharing resources identified through application of NLP to CORD-19 database

No.	Title of citation for potential tool	Assessment of utility as resource for collecting, harmonizing and sharing COVID-19 participant-level data
1	OutbreakTools: A new platform for disease outbreak analysis using the R software	Does not collect, harmonize, share COVID-19 participant-level data
2	Erratum: A biomimetic hybrid nanoplatform for encapsulation and precisely controlled delivery of theranostic agents	Does not collect, harmonize, share COVID-19 participant-level data
3	Corrigendum: A coral-on-a-chip microfluidic platform enabling live-imaging microscopy of reef-building corals	Does not collect, harmonize, share COVID-19 participant-level data
4	Project OPUS: Development and evaluation of an electronic platform for pain management education of medical undergraduates in resource-limited settings	Does not collect, harmonize, share COVID-19 participant-level data
5	A Self-Assembling Ferritin Nanoplatform for Designing Classical Swine Fever Vaccine: Elicitation of Potent Neutralizing Antibody	Does not collect, harmonize, share COVID-19 participant-level data
6	Amikacin pharmacokinetic/pharmacodynamic in intensive care unit: a prospective database	Does not collect, harmonize, share COVID-19 participant-level data
7	An Optimizing Multi-platform Source-to-source Compiler Framework for the NEURON MODeling Language	Does not collect, harmonize, share COVID-19 participant-level data
8	LitCovid: an open database of COVID-19 literature	Does not collect, harmonize, share COVID-19 participant-level data
9	DRDOCK: A drug repurposing platform integrating automated docking, simulations and a log-odds-based drug ranking scheme	Does not collect, harmonize, share COVID-19 participant-level data
10	Trialstreamer: a living, automatically updated database of clinical trial reports	Does not collect, harmonize, share COVID-19 participant-level data
11	REPP: A robust cross-platform solution for online sensorimotor synchronization experiments	Does not collect, harmonize, share COVID-19 participant-level data
12	SARS Grid--an AG-based disease management and collaborative platform	Does not collect, harmonize, share COVID-19 participant-level data
13	COVIDScholar: An automated COVID-19 research aggregation and analysis platform	Does not collect, harmonize, share COVID-19 participant-level data
14	LitCovid: an open database of COVID-19 literature	Duplicate (8)
15	DBCOVP: A database of coronavirus virulent glycoproteins	Does not collect, harmonize, share COVID-19 participant-level data

No.	Title of citation for potential tool	Assessment of utility as resource for collecting, harmonizing and sharing COVID-19 participant-level data
16	Telehealth Training During the COVID-19 Pandemic: A Feasibility Study of Large Group Multiplatform Telesimulation Training	Does not collect, harmonize, share COVID-19 participant-level data
17	Nanoplatforms for mRNA Therapeutics	Does not collect, harmonize, share COVID-19 participant-level data
18	Covid-19 Disease Simulation using GAMA platform	Does not collect, harmonize, share COVID-19 participant-level data
19	BioDynaMo: a general platform for scalable agent-based simulation	Does not collect, harmonize, share COVID-19 participant-level data
20	COVID-19 Disease Map, building a computational repository of SARS-CoV-2 virus-host interaction mechanisms	Does not collect, harmonize, share COVID-19 participant-level data
21	ERACODA: the European database collecting clinical information of patients on kidney replacement therapy with COVID-19	Registry that collects participant-level longitudinal clin-epi data about patients on kidney replacement therapy with COVID-19, harmonizes the data, but does not share the participant-level data
22	The National Gene Vector Biorepository's Pharm/Tox Database	Does not collect, harmonize, share COVID-19 participant-level data
23	MMDB: annotating protein sequences with Entrez's 3D-structure database	Does not collect, harmonize, share COVID-19 participant-level data
24	FSDB: A frameshift signal database	Does not collect, harmonize, share COVID-19 participant-level data
25	National Sample Vital Registration System: A sustainable platform for COVID-19 and other infectious diseases surveillance in low and middle-income countries	Does not collect, harmonize, share COVID-19 participant-level data
26	Virus-CKB: an integrated bioinformatics platform and analysis resource for COVID-19 research	Does not collect, harmonize, share COVID-19 participant-level data
27	Pathosphere.org: pathogen detection and characterization through a web-based, open source informatics platform	Does not collect, harmonize, share COVID-19 participant-level data (website is not available)
28	RNAcentral 2021: secondary structure integration, improved sequence search and new member databases	Does not collect, harmonize, share COVID-19 participant-level data
29	Infectome: A platform to trace infectious triggers of autoimmunity	Does not collect, harmonize, share COVID-19 participant-level data
30	CoVDB: a comprehensive database for comparative analysis of coronavirus genes and genomes	Does not collect, harmonize, share COVID-19 participant-level data
31	SARS-CoV-2 RECoVERY: A multi-platform open-source bioinformatic pipeline for the automatic construction and analysis of SARS-CoV-2 genomes from NGS sequencing data	Does not collect, harmonize, share COVID-19 participant-level data

No.	Title of citation for potential tool	Assessment of utility as resource for collecting, harmonizing and sharing COVID-19 participant-level data
32	The de.NBI / ELIXIR-DE training platform - Bioinformatics training in Germany and across Europe within ELIXIR	Does not collect, harmonize, share COVID-19 participant-level data
33	McQ - An open-source multiplexed SARS-CoV-2 quantification platform	Does not collect, harmonize, share COVID-19 participant-level data
34	Architectured Therapeutic and Diagnostic Nanoplatforms for Combating SARS-CoV-2: Role of Inorganic, Organic, and Radioactive Materials	Does not collect, harmonize, share COVID-19 participant-level data
35	Covid19db: An online database of trials of medicinal products to prevent or treat COVID-19, with a specific focus on drug repurposing	Does not collect, harmonize, share COVID-19 participant-level data
36	DatAC: A visual analytics platform to explore climate and air quality indicators associated with the COVID-19 pandemic in Spain	Does not collect, harmonize, share COVID-19 participant-level data
37	VIDA: a virus database system for the organization of animal virus genome open reading frames	Does not collect, harmonize, share COVID-19 participant-level data
38	MoonProt 3.0: an update of the moonlighting proteins database	Does not collect, harmonize, share COVID-19 participant-level data
39	CVTree update: a newly designed phylogenetic study platform using composition vectors and whole genomes	Does not collect, harmonize, share COVID-19 participant-level data
40	PhEVER: a database for the global exploration of virus–host evolutionary relationships	Does not collect, harmonize, share COVID-19 participant-level data
41	ELM—the database of eukaryotic linear motifs	Does not collect, harmonize, share COVID-19 participant-level data
42	IMG/VR: a database of cultured and uncultured DNA Viruses and retroviruses	Does not collect, harmonize, share COVID-19 participant-level data
43	SPRINT: a Cas13a-based platform for detection of small molecules	Does not collect, harmonize, share COVID-19 participant-level data
44	BiteOscope, an open platform to study mosquito biting behavior	Does not collect, harmonize, share COVID-19 participant-level data
45	Publisher Correction: Image Data Resource: a bioimage data integration and publication platform	Does not collect, harmonize, share COVID-19 participant-level data
46	Trialstreamer: A living, automatically updated database of clinical trial reports	Duplicate (10)
47	ThermoMutDB: a thermodynamic database for missense mutations	Does not collect, harmonize, share COVID-19 participant-level data
48	CEN-tools: an integrative platform to identify the contexts of essential genes	Does not collect, harmonize, share COVID-19 participant-level data

No.	Title of citation for potential tool	Assessment of utility as resource for collecting, harmonizing and sharing COVID-19 participant-level data
49	Early results of the Axium MicroFX for Endovascular Repair of IntraCranial Aneurysm (AMERICA) study: a multicenter prospective observational registry	Does not collect, harmonize, share COVID-19 participant-level data
50	CustusX: an open-source research platform for image-guided therapy	Does not collect, harmonize, share COVID-19 participant-level data
51	Neurologic manifestations in hospitalized patients with COVID-19: The ALBACOVID registry	Does not collect, harmonize, share COVID-19 participant-level data (closed study)
52	icumonitoring.ch: a platform for short-term forecasting of intensive care unit occupancy during the COVID-19 epidemic in Switzerland	Does not collect, harmonize, share COVID-19 participant-level data
53	[TCMATCOV--a bioinformatics platform to predict efficacy of TCM against COVID-19]	Does not collect, harmonize, share COVID-19 participant-level data
54	Cellinker: a platform of ligand-receptor interactions for intercellular communication analysis	Does not collect, harmonize, share COVID-19 participant-level data
55	SECURE-Psoriasis: A de-identified registry of psoriasis patients diagnosed with COVID-19	Registry that collects participant-level cross-sectional clin-epi data about patients with psoriasis and COVID-19, harmonizes the data, but does not share the participant-level data
56	CMAUP: a database of collective molecular activities of useful plants	Does not collect, harmonize, share COVID-19 participant-level data
57	Commentary: The MISAGO registry: a rapid-exchange superficial femoral artery stent for a rapidly expanding field	Does not collect, harmonize, share COVID-19 participant-level data
58	PolarProtDb: a database of transmembrane and secreted proteins showing apical-basal polarity	Does not collect, harmonize, share COVID-19 participant-level data
59	SILK flow diverter for complex intracranial aneurysms: a Canadian registry	Does not collect, harmonize, share COVID-19 participant-level data
60	LncExpDB: an expression database of human long non-coding RNAs	Does not collect, harmonize, share COVID-19 participant-level data
61	Rapidemic, a versatile and label-free DNAzyme-based platform for visual nucleic acid detection	Does not collect, harmonize, share COVID-19 participant-level data
62	Donut PCR: a rapid, portable, multiplexed, and quantitative DNA detection platform with single-nucleotide specificity	Does not collect, harmonize, share COVID-19 participant-level data
63	DREIMT: a drug repositioning database and prioritization tool for immunomodulation	Does not collect, harmonize, share COVID-19 participant-level data
64	Comprehensive mapping of local and diaspora scientists: a database and analysis of 63951 Greek scientists	Does not collect, harmonize, share COVID-19 participant-level data

No.	Title of citation for potential tool	Assessment of utility as resource for collecting, harmonizing and sharing COVID-19 participant-level data
65	PFDB: a generic protein family database integrating the CATH domain structure database with sequence based protein family resources	Does not collect, harmonize, share COVID-19 participant-level data
66	COVID-19 management in heart transplanted recipients: registry of Almazov National Medical Research Centre	Does not collect, harmonize, share COVID-19 participant-level data (closed study)
67	Ubiquitous Health Profile (UHPr): a big data curation platform for supporting health data interoperability	Does not collect, harmonize, share COVID-19 participant-level data
68	WiFiMon: A mobility analytics platform for building occupancy monitoring and contact tracing using wifi sensing: Poster abstract	Does not collect, harmonize, share COVID-19 participant-level data
69	List N: Disinfectants for Use Against SARS-CoV-2 [database]	Does not collect, harmonize, share COVID-19 participant-level data
70	Neurologic manifestations associated with COVID-19: a multicentre registry	Registry that collects participant-level longitudinal clin-epi data about patients with neurological conditions and COVID-19, harmonizes the data, and shares this data. Has several prospective cohorts including adult and pediatric cohorts
71	Propedia: a database for protein-peptide identification based on a hybrid clustering algorithm	Does not collect, harmonize, share COVID-19 participant-level data
72	icumonitoring.ch: a platform for short-term forecasting of intensive care unit occupancy during the COVID-19 epidemic in Switzerland	Duplicate (52)
73	[TCMATCOV--a bioinformatics platform to predict efficacy of TCM against COVID-19]	Duplicate (53)
74	Cellinker: a platform of ligand-receptor interactions for intercellular communication analysis	Duplicate (54)
75	Covigie, a platform for caregivers and care team coordinators	Does not collect, harmonize, share COVID-19 participant-level data
76	SECURE-Psoriasis: a de-identified registry of psoriasis patients diagnosed with COVID-19	Duplicate (55)
77	Global Hidradenitis Suppurativa COVID-19 Registry: a registry to inform data-driven management practices	Registry that collects participant-level cross-sectional clin-epi data about patients with hidradenitis suppurativa and COVID-19, harmonizes the data, but does not share the participant-level data
78	COVID-19 pandemic: Coroner's database of death inquiries with clinical epidemiology and total and excess mortality analyses in the District of Kildare March to June 2020	Does not collect, harmonize, share COVID-19 participant-level data
79	AgAcademy: a modal platform for scaling up e-learning in Indian agriculture in COVID times	Does not collect, harmonize, share COVID-19 participant-level data

No.	Title of citation for potential tool	Assessment of utility as resource for collecting, harmonizing and sharing COVID-19 participant-level data
80	CGAP: a new comprehensive platform for the comparative analysis of chloroplast genomes	Does not collect, harmonize, share COVID-19 participant-level data
81	METAGENOTE: a simplified web platform for metadata annotation of genomic samples and streamlined submission to NCBI's sequence read archive	Does not collect, harmonize, share COVID-19 participant-level data
82	ViPR: an open bioinformatics database and analysis resource for virology research	Does not collect, harmonize, share COVID-19 participant-level data
83	outbreaker2: a modular platform for outbreak reconstruction	Does not collect, harmonize, share COVID-19 participant-level data
84	MRPrimerV: a database of PCR primers for RNA virus detection	Does not collect, harmonize, share COVID-19 participant-level data
85	Anticovid, a comprehensive open-access real-time platform of registered clinical studies for COVID-19	Does not collect, harmonize, share COVID-19 participant-level data
86	The Brighton Collaboration standardized template for collection of key information for risk/benefit assessment of a Modified Vaccinia Ankara (MVA) vaccine platform	Does not collect, harmonize, share COVID-19 participant-level data
87	COVID-19 and its sequelae: a platform for optimal patient care, discovery and training	Does not collect, harmonize, share COVID-19 participant-level data
88	Reference sequence (RefSeq) database at NCBI: current status, taxonomic expansion, and functional annotation	Does not collect, harmonize, share COVID-19 participant-level data
89	FragMAX: the fragment-screening platform at the MAX IV Laboratory	Does not collect, harmonize, share COVID-19 participant-level data
90	opvCRISPR: One-pot visual RT-LAMP-CRISPR platform for SARS-cov-2 detection	Does not collect, harmonize, share COVID-19 participant-level data
91	PURY: a database of geometric restraints of hetero compounds for refinement in complexes with macromolecular structures	Does not collect, harmonize, share COVID-19 participant-level data
92	HIT-COVID, a global database tracking public health interventions to COVID-19	Does not collect, harmonize, share COVID-19 participant-level data
93	A collection of designed peptides to target SARS-Cov-2 – ACE2 interaction: PepI-Covid19 database	Does not collect, harmonize, share COVID-19 participant-level data
94	SPDB: a specialized database and web-based analysis platform for swine pathogens	Does not collect, harmonize, share COVID-19 participant-level data
95	MarkerDB: an online database of molecular biomarkers	Does not collect, harmonize, share COVID-19 participant-level data

No.	Title of citation for potential tool	Assessment of utility as resource for collecting, harmonizing and sharing COVID-19 participant-level data
96	ROBOCOV: An affordable open-source robotic platform for COVID-19 testing by RT-qPCR	Does not collect, harmonize, share COVID-19 participant-level data
97	Neurological manifestations associated with COVID-19: a multicentric registry	Duplicate (70)
98	The spectrum of COVID-19-associated dermatologic manifestations: an international registry of 716 patients from 31 countries	Registry that collects participant-level cross-sectional clin-epi data about patients with dermatologic conditions and COVID-19, harmonizes the data, and shares this data
99	ADPriboDB 2.0: an updated database of ADP-ribosylated proteins	Does not collect, harmonize, share COVID-19 participant-level data
100	COVID-19 Disease Map, a computational knowledge repository of SARS-CoV-2 virus-host interaction mechanisms	Does not collect, harmonize, share COVID-19 participant-level data
101	RAPPID: a platform of ratiometric bioluminescent sensors for homogeneous immunoassays	Does not collect, harmonize, share COVID-19 participant-level data
102	DockCoV2: a drug database against SARS-CoV-2	Does not collect, harmonize, share COVID-19 participant-level data
103	Covid19Risk.ai: An open source repository and online calculator of prediction models for early diagnosis and prognosis of Covid-19	Does not collect, harmonize, share COVID-19 participant-level data
104	Virus taxonomy: the database of the International Committee on Taxonomy of Viruses (ICTV)	Does not collect, harmonize, share COVID-19 participant-level data
105	COVIDep: a web-based platform for real-time reporting of vaccine target recommendations for SARS-CoV-2	Does not collect, harmonize, share COVID-19 participant-level data
106	A2A: a platform for research in biomedical literature search	Does not collect, harmonize, share COVID-19 participant-level data
107	DPL: a comprehensive database on sequences, structures, sources and functions of peptide ligands	Does not collect, harmonize, share COVID-19 participant-level data
108	ViruSurf: an integrated database to investigate viral sequences	Does not collect, harmonize, share COVID-19 participant-level data
109	DescribePROT: database of amino acid-level protein structure and function predictions	Does not collect, harmonize, share COVID-19 participant-level data
110	CoV3D: a database of high resolution coronavirus protein structures	Does not collect, harmonize, share COVID-19 participant-level data
111	CORDITE: the curated CORona Drug InTERactions database for SARS-CoV-2	Does not collect, harmonize, share COVID-19 participant-level data
112	Pfam: The protein families database in 2021	Does not collect, harmonize, share COVID-19 participant-level data
113	Propedia: a database for protein–peptide identification based on a hybrid clustering algorithm	Duplicate (71)

No.	Title of citation for potential tool	Assessment of utility as resource for collecting, harmonizing and sharing COVID-19 participant-level data
114	AlzGPS: a genome-wide positioning systems platform to catalyze multi-omics for Alzheimer's drug discovery	Does not collect, harmonize, share COVID-19 participant-level data
115	Swab-Seq: A high-throughput platform for massively scaled up SARS-CoV-2 testing	Does not collect, harmonize, share COVID-19 participant-level data
116	Engineering organoids: a promising platform to understand biology and treat diseases	Does not collect, harmonize, share COVID-19 participant-level data
117	Aging Atlas: a multi-omics database for aging biology	Does not collect, harmonize, share COVID-19 participant-level data
118	COVeAGE-DB: A database of age-structured COVID-19 cases and deaths	Does not collect, harmonize, share COVID-19 participant-level data
119	Validation of the Provincial Transfer Authorization Centre database: a comprehensive database containing records of all inter-facility patient transfers in the province of Ontario	Does not collect, harmonize, share COVID-19 participant-level data
120	COVID-19 Variants Database: A repository for Human SARS-CoV-2 Polymorphism Data	Tool that obtains data from the National Genomics Data Center (NGDC), Global Initiative on Sharing All Influenza Data (GISAID), and National Center for Biotechnology Information (NCBI) Genbank to help visualize the variants in the SARS-CoV-2 viral genome
121	OxCOVID19 Database: a multimodal data repository for better understanding the global impact of COVID-19	Does not collect, harmonize, share COVID-19 participant-level data
122	VIRsiRNAdb: a curated database of experimentally validated viral siRNA/shRNA	Does not collect, harmonize, share COVID-19 participant-level data
123	Guide to Immunopharmacology: a database to boost immunology education, research and therapy	Does not collect, harmonize, share COVID-19 participant-level data
124	GESS: a database of global evaluation of SARS-CoV-2/hCoV-19 sequences	Platform that obtains data from GISAID that allows users to browse, search and download single nucleotide variants at any individual or multiple SARS-CoV-2 genomic positions, or within a chosen genomic region or protein, or in a certain country/area of interest
125	DBatVir: the database of bat-associated viruses	Does not collect, harmonize, share COVID-19 participant-level data
126	H2V: a database of human genes and proteins that respond to SARS-CoV-2, SARS-CoV, and MERS-CoV infection	Does not collect, harmonize, share COVID-19 participant-level data
127	SARS2020: An integrated platform for identification of novel coronavirus by a consensus sequence-function model	Does not collect, harmonize, share COVID-19 participant-level data

No.	Title of citation for potential tool	Assessment of utility as resource for collecting, harmonizing and sharing COVID-19 participant-level data
128	The baculovirus expression vector system: A commercial manufacturing platform for viral vaccines and gene therapy vectors	Does not collect, harmonize, share COVID-19 participant-level data
129	CAPACITY-COVID: a European registry to determine the role of cardiovascular disease in the COVID-19 pandemic	Registry that collects participant-level longitudinal clin-epi data about patients with cardiovascular complications and COVID-19, harmonizes the data, and shares this data
130	Viral nanoparticles and virus-like particles: platforms for contemporary vaccine design	Does not collect, harmonize, share COVID-19 participant-level data
131	VirOligo: a database of virus-specific oligonucleotides	Does not collect, harmonize, share COVID-19 participant-level data
132	AVPdb: a database of experimentally validated antiviral peptides targeting medically important viruses	Does not collect, harmonize, share COVID-19 participant-level data

Supplementary Figure 1. Registry and platform-specific linkages between data types

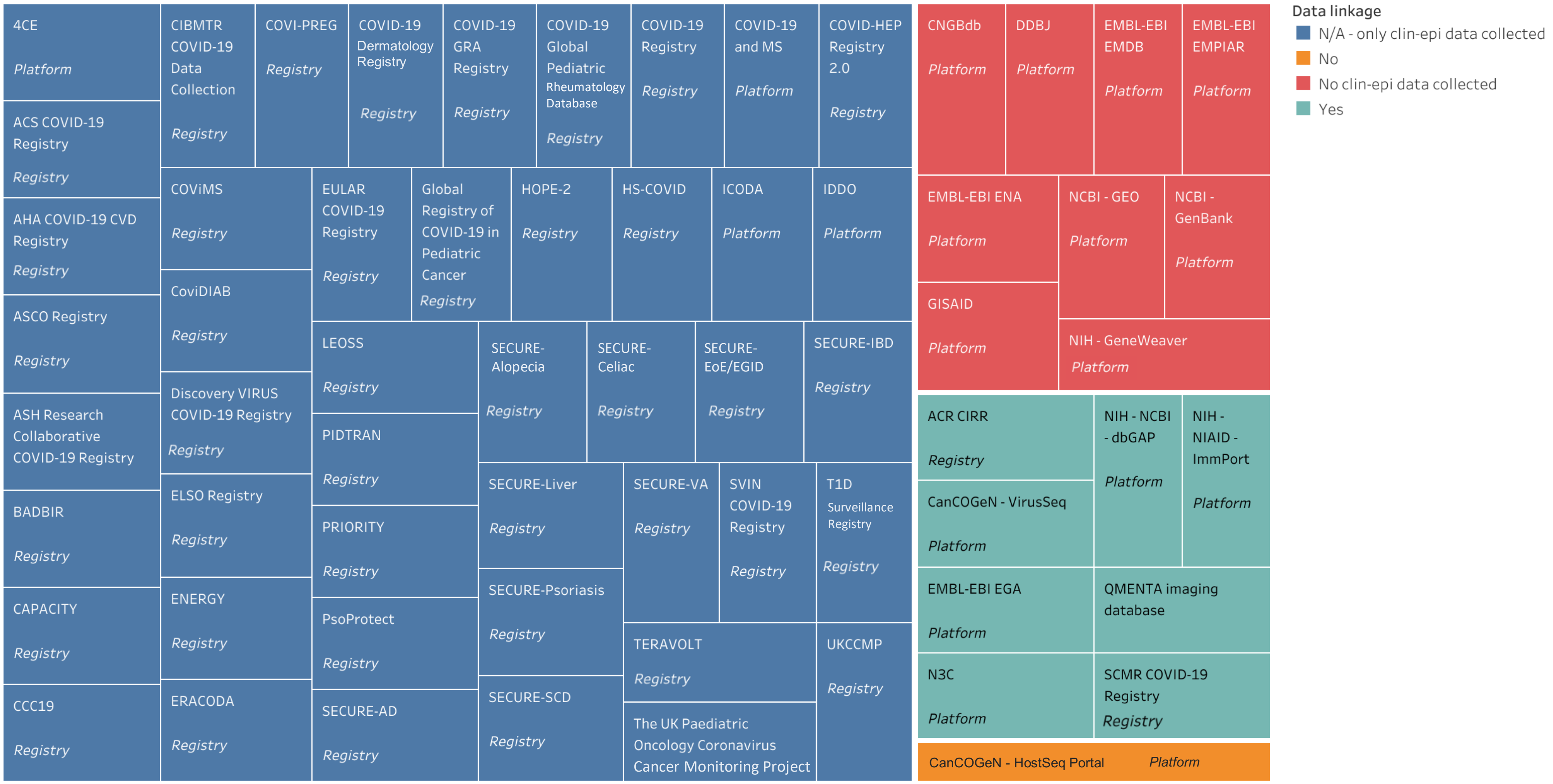

Supplementary Figure 2. Summary distribution of platforms and registries for sharing participant-level COVID-19-related health data*

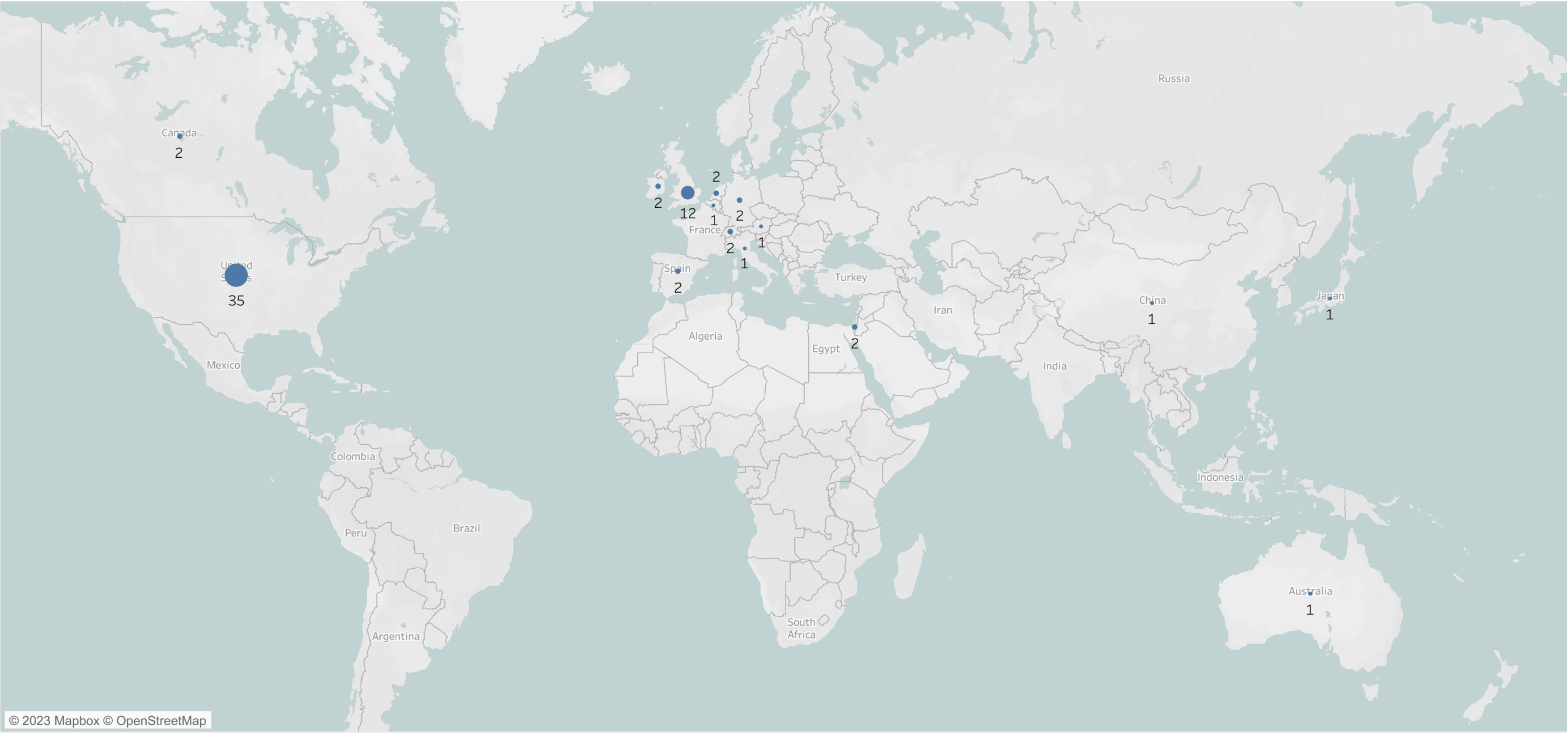

*Circle size is proportional to the number of registries or platforms for collecting, harmonizing, sharing COVID-19.

Supplementary Figure 3. Distribution of platforms and registries for sharing participant-level COVID-19-related health data

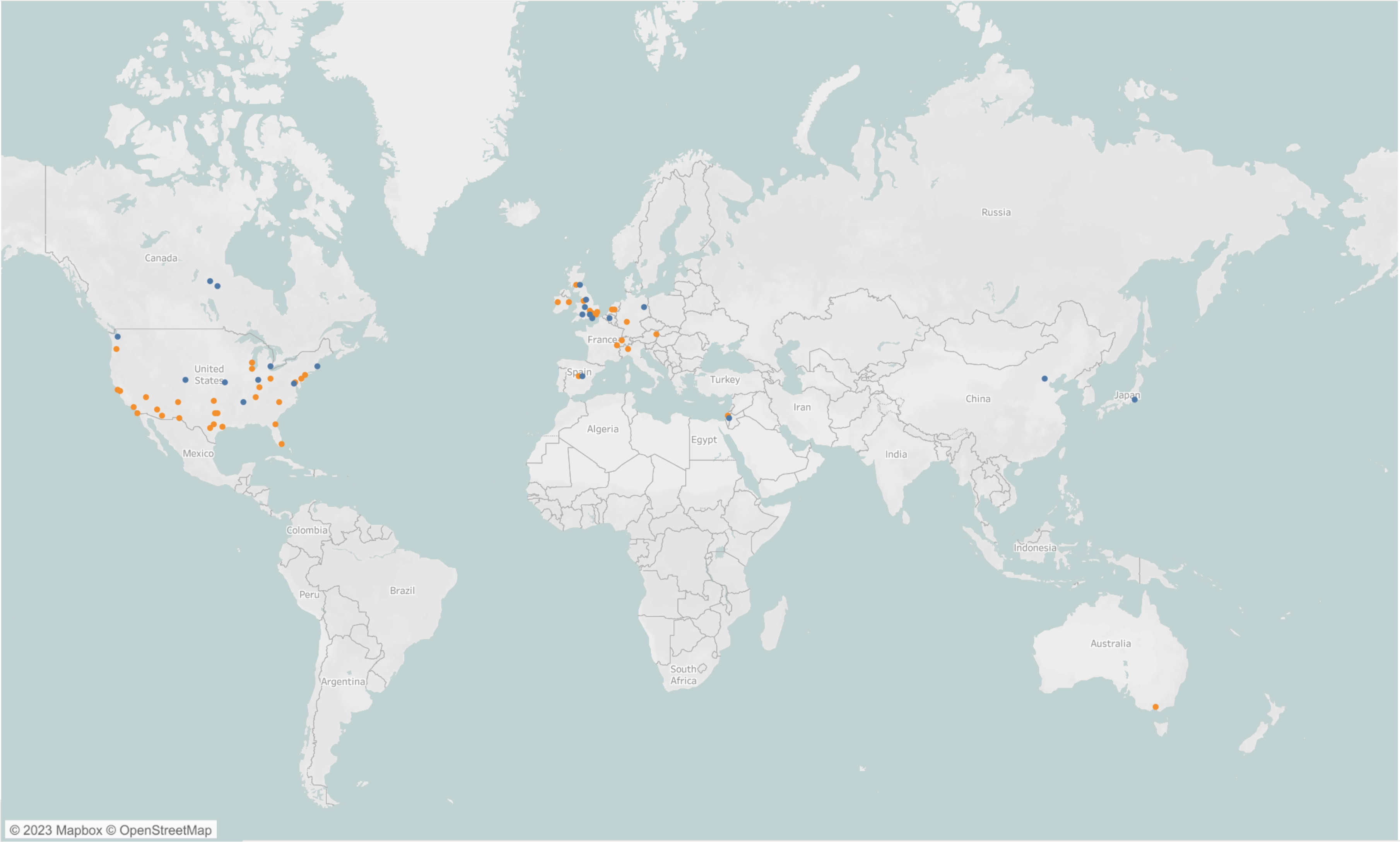

*Blue circles represent the location of platforms. Orange circles represent the location of registries.

Supplementary Table 4. Summary of principles from health data sharing frameworks

General domain	7 GloPID-R Principles of Sharing Data in Public Health Emergencies[13]	COVID-19 NCS Data Sharing Principles[14]	International Code of Conduct for Data Sharing in Genomic Research[15]	GA4GH Framework for Responsible Sharing of Genomic and Health Related Data[16]	CARE Principles for Indigenous Data Governance[17]
Collaboration		Work collaboratively to actively share data to allow the scientific community to pool expertise, draw fresh insights, increase collective understanding.	Responsibility: Responsible governance should be shared between funders, generators and users of data. Investments in databases require coordination, strategy and long-term core funding. Mechanisms for building interoperability should be encouraged and appropriate management anticipated. Capacity building and recognition of all the data generators contributes to best practice.	Education & training: Dedicate education and training resources so as to advance data sharing and data management and to constantly improve data quality and integrity.	
FAIR data	Accessible: Data pertaining to PHEs should be shared with as few restrictions, either technical or legal, as possible. Providers of data should clearly indicate what, if any, conditions are in place, and for how long they apply.<br>FAIRness: The provision and use of data must be done in such a way that ensures fair treatment of all parties involved and recognition of their contributions. Further, any use of data should respect and acknowledge the provider and/or origin of the data and terms under which that data can be accessed and should reflect international commitments to benefits sharing.	Ensure all data and associated code and tools generated through the studies are FAIR. Make research outputs, observations, code and tools generated from the studies open-source, rapidly and freely accessible as a public good.	Accessible: Facilitation of both the deposit of data and secure access to data are the foundations of data sharing. Curators of databases should promote sharing to generate maximum value. Harmonization of deposit, access procedures and use promotes accessibility, equity and transparency.		
Ethical	Sharing of data must be done in accordance with applicable ethical and legal standards, ensuring beneficence and respect for confidentiality, the privacy of individuals and the dignity of communities. This is essential for building the trust of the public and	Consent: Ensure unconsented data is accessed through secure platforms accredited or working towards accreditation by the UK Statistics Authority to comply standards established according to Digital Economy Act requirements, or working towards	Integrity: Mutual respect between all stakeholders is founded on personal and professional integrity. Prevention of harms and anticipation of public concerns and scientific needs through foresight mechanisms encourage the development of common,	Risk-Benefit Analysis: Consider the realistic harms and benefits of data sharing on and with individuals, families and communities, including opportunity costs associated with both sharing and not sharing data. Conduct data sharing with a view towards minimizing harms and maximizing benefits to not just	All data sharing should protect the privacy of individuals and the dignity of communities, while simultaneously respecting the imperative to improve public health through the

General domain	7 GloPID-R Principles of Sharing Data in Public Health Emergencies[13]	COVID-19 NCS Data Sharing Principles[14]	International Code of Conduct for Data Sharing in Genomic Research[15]	GA4GH Framework for Responsible Sharing of Genomic and Health Related Data[16]	CARE Principles for Indigenous Data Governance[17]
	all stakeholders. Additional attention should be given to respect for, and alignment with, cultural norms.	this status, which allows insights to be generated whilst maintaining privacy and data security.	prospective policies. Sanctions for breach of this Code or of other legal or ethical obligations must be clear.	those who contribute their data, but also to society and health care systems as a whole. Security: Establish proportionate data security measures that mitigate the risk of unauthorized access, data loss and misuse.	most productive use of data.
Community engagement	Equitable: Data should be made available to all interested parties during a PHE at no cost, or at a cost recovery level only. This approach will help to ensure that all parties, including data providers and data users, have equal access to the data needed to collaborate and collectively deliver benefits to communities affected by a health emergency.	Demonstrate active and ongoing engagement with patients and the public in the design, development and governance of their activities, to provide assurance that these activities are in the public interest.	Accountability: Inter-agency co-operation and funding fosters streamlined and efficient monitoring and good governance. Provisions should be made for ongoing public engagement that is tailored to the nature of the database and local cultures.		Equitable: Any approach to the sharing of data should recognise and balance the needs of researchers who generate and use data, other analysts who might want to reuse those data, and communities and funders who expect health benefits to arise from research.
Transparent governance	The process for sharing data and facilitating access should be clearly explained, outlining how and when the data can and cannot be shared and defining the associated descriptors of the data.	Be transparent in the use of personal data and respect the privacy and confidentiality of individuals, complying with legal requirements and ethical expectations at all times.	Key policies on publications, intellectual property, and industry involvement should be public. Websites that are accessible to the general public serve to provide feedback on progress and general results.	Develop clearly defined and accessible information on the purposes, processes, procedures and governance frameworks for data sharing.	
Compliance with data protection laws		Be transparent in the use of personal data and respect the privacy and confidentiality of individuals, complying with legal requirements and ethical expectations at all times.	Security: Trust and the promotion of data sharing rely on data management and security mechanisms and also on oversight of their functioning. Mechanisms for identifying and tracking data generators and users should be international.	Privacy, Data protection, Confidentiality: Comply with applicable privacy and data protection regulations at every stage of data sharing.	
Evaluate platform utility		Demonstrate value for money by using existing UK infrastructure and research investments as far as possible and using open competitions where necessary to develop new infrastructure capability.		Accountability: Put in place systems for data sharing that respect this Framework.Track the chain of data access and/or exchange to its source. Develop processes to identify and manage conflicts of interest. Implement mechanisms for handling complaints related to data misuse; for identifying, reporting and	Efficient: Any approach to data sharing should improve the quality and value of research and increase its contribution to improving public health. Approaches should be proportionate and build on

General domain	7 GloPID-R Principles of Sharing Data in Public Health Emergencies[13]	COVID-19 NCS Data Sharing Principles[14]	International Code of Conduct for Data Sharing in Genomic Research[15]	GA4GH Framework for Responsible Sharing of Genomic and Health Related Data[16]	CARE Principles for Indigenous Data Governance[17]
				managing breaches; and for instituting appropriate sanctions.	existing practice and reduce unnecessary duplication and competition.
Quality	The minimum quality standard of data must be ensured by the provider while data users must also ensure that data processing, analysis and interpretation are conducted with an equal or greater application of quality standards. Appropriate and recognised data standards should be adhered to, while all relevant metadata, methodology, assumptions and experimental details should be provided with the data. This will ensure that any work conducted from the data takes into account the context in which the data was originally produced.		Irrespective of the discipline, scientists involved in data sharing should be *bona fide* researchers. Proof of academic or other recognized peer reviewed standing is essential. Harmonization of data collection and archiving methods and tools ensures validation of scientific quality. Collaboration promotes efficiency, sustainability and comparability.	Data quality & security: Store and process the data collected, used and transferred in a way that is accurate, verifiable, unbiased, proportionate, and current, so as to enhance their interoperability and replicability and also preserve their long-term searchability and integrity. Ensure feedback mechanisms on the utility, quality, security, and accuracy of data, and their annotations, with a view to improving quality and interoperability and appropriate re-use by others.	

CARE=Collective benefit, Authority to control, Responsibility, Ethics. GA4GH=Global Alliance for Genomics and Health. GloPID-R, Global Research Collaboration for Infectious Disease Preparedness. NCS=National Core Studies.

Supplementary Table 5. Platforms and registries associated with Figure 1

	General	Specific coinfection, comorbidity, assessment, treatment, outcome
**FAIR enough**	Platforms:<br>• Canadian COVID-19 Genomics Network - VirusSeq Data Portal (CanCOGeN-VirusSeq)<br>• China National GeneBank DataBase (CNGBdb)<br>• DNA Data Bank of Japan (DDBJ)<br>• Electron Microscopy Data Bank (EMDB)<br>• Electron Microscopy Public Image Archive (EMPIAR)<br>• European Genome-phenome Archive (EGA)<br>• European Nucleotide Archive (ENA)<br>• Genbank<br>• Gene Expression Omnibus (GEO)<br>• GeneWeaver<br>• Global Initiative on Sharing All Influenza Data (GISAID)<br>• Infectious Diseases Data Observatory (IDDO)<br>• International COVID-19 Data Alliance (ICODA)<br>• National COVID Cohort Collaborative (N3C)<br>• The Consortium for Clinical Characterization of COVID-19 by EHR (4CE)<br>• The database of Genotypes and Phenotypes (dbGaP)<br>• The Immunology Database and Analysis Portal (ImmPort)<br><br>Registries:<br>• Lean European Open Survey on SARS-CoV-2 (LEOSS)	Platforms:<br>• QMENTA imaging database<br>Registries:<br>• American Heart Association (AHA) COVID-19 Cardiovascular Disease Registry<br>• American Society of Clinical Oncology (ASCO) Survey on COVID-19 in Oncology Registry<br>• British Association of Dermatologists Biologic and Immunomodulators Register (BADBIR)<br>• Cardiac Complications in Patients with SARS Corona virus 2 registry (CAPACITY)<br>• Center for International Blood and Marrow Transplant Research (CIBMTR) COVID-19 Data Collection<br>• COVID-19 Dermatology registry<br>• COVID-19 Global Pediatric Rheumatology Database<br>• COVID-Hepatology Registry<br>• Discovery Viral Infection and Respiratory Illness Universal Study COVID-19 Registry<br>• Extracorporeal Life Support Organization (ELSO) Registry<br>• Pediatric COVID-19 Case Registry (PIDTRAN)<br>• Psoriasis Patient Registry for Outcomes, Therapy and Epidemiology of COVID-19 Infection (PsoProtect)<br>• Society for Cardiovascular Magnetic Resonance (SCMR) COVID-19 Registry<br>• Surveillance Epidemiology of Coronavirus Under Research Exclusion-Inflammatory Bowel Disease (SECURE-IBD)<br>• The COVID-19 and Cancer Consortium (CCC19)<br>• The European Renal Association COVID-19 Database (ERACODA)

**Not very FAIR**	Platforms:<br>• CanCOGeN - HostSeq Portal<br><br>Registries:<br>• COVID-19 Registry	Platforms:<br>• COVID-19 and MS – a global data sharing initiative<br><br>Registries:<br>• American College of Radiology COVID-19 Imaging Research Registry (ACR CIRR)<br>• American College of Surgeons (ACS) COVID-19 Registry<br>• American Society for Hematology (ASH) Research Collaborative COVID-19 Registry for Hematologic Malignancy<br>• Coronavirus and MS Reporting Database (COViMS)<br>• European Academy of Neurology Neuro-covid Registry (ENERGY)<br>• Global Hidradenitis Suppurativa COVID-19 Registry (HS-COVID)<br>• Global Registry of COVID-19 in Pediatric Cancer<br>• Global Registry of COVID-19-related Diabetes (CoviDIAB)<br>• Health Outcome Predictive Evaluation for COVID 19-2 (HOPE-2)<br>• International COVID-19 and Pregnancy Registry (COVI-PREG)<br>• Pregnancy Coronavirus Outcomes Registry (PRIORITY)<br>• SECURE-Alopecia<br>• SECURE-Atopic Dermatitis (SECURE-AD)<br>• SECURE-Celiac<br>• SECURE-Eosinophilic Esophagitis (EoE) and Eosinophilic Gastrointestinal Diseases (EGID) (SECURE-EoE/EGID)<br>• SECURE-Liver<br>• SECURE-Psoriasis<br>• SECURE-Sickle Cell Disease (SECURE-SCD)<br>• SECURE-vascular anomalies (SECURE-VA)

		• Society of Vascular and Interventional Neurology (SVIN) COVID-19 Registry<br>• The COVID-19 Global Rheumatology Alliance Registry (COVID-19 GRA Registry)<br>• The European Alliance of Associations for Rheumatology (EULAR) COVID-19 Registry<br>• The UK Coronavirus Cancer Monitoring Project (UKCCMP)<br>• The UK Paediatric Oncology Coronavirus Cancer Monitoring Project<br>• Thoracic Cancers International COVID-19 Collaboration Registry (TERAVOLT)<br>• Type 1 Diabetes COVID-19 multi-site surveillance registry
	**FAIR**	**Not very FAIR**
**Clin-epi data only**	Platforms:<br>• 4CE<br>• ICODA<br>• IDDO<br><br>Registries:<br>• AHA COVID-19 Cardiovascular Disease Registry<br>• ASCO Survey on COVID-19 in Oncology Registry<br>• BADBIR<br>• CAPACITY<br>• CCC19<br>• CIBMTR COVID-19 Data Collection<br>• COVID-19 Dermatology registry<br>• COVID-19 Global Pediatric Rheumatology Database<br>• COVID-Hepatology Registry<br>• Discovery Viral Infection and Respiratory Illness Universal Study COVID-19 Registry<br>• ELSO Registry<br>• ERACODA	Platforms:<br>• COVID-19 and MS – a global data sharing initiative<br><br>Registries:<br>• ACS COVID-19 Registry<br>• ASH Research Collaborative COVID-19 Registry for Hematologic Malignancy<br>• COVI-PREG<br>• COVID-19 GRA Registry<br>• COVID-19 Registry<br>• CoviDIAB<br>• COViMS<br>• ENERGY<br>• EULAR COVID-19 Registry<br>• Global Registry of COVID-19 in Pediatric Cancer<br>• HOPE-2<br>• HS-COVID<br>• PRIORITY<br>• SECURE-AD

	• LEOSS<br>• PIDTRAN<br>• PsoProtect	• SECURE-Alopecia<br>• SECURE-Celiac<br>• SECURE-EoE/EGID<br>• SECURE-Liver<br>• SECURE-Psoriasis<br>• SECURE-SCD<br>• SECURE-VA<br>• SVIN COVID-19 Registry<br>• TERAVOLT<br>• The UK Paediatric Oncology Coronavirus Cancer Monitoring Project<br>• Type 1 Diabetes COVID-19 multi-site surveillance registry<br>• UKCCMP
**Human OMICs data only**	Platforms:<br>• GeneWeaver<br><br>Registries:<br>None	Platforms:<br>None<br><br>Registries:<br>None
**Imaging data only**	Platforms:<br>• EMDB<br>• EMPIAR<br><br>Registries:<br>None	Platforms:<br>None<br><br>Registries:<br>None
**>1 data type**	Platforms:<br>• CanCOGeN-VirusSeq<br>• dbGaP<br>• DDBJ<br>• EGA<br>• ENA<br>• GenBank<br>• GEO	Platforms:<br>• CanCOGeN - HostSeq Portal<br><br>Registries:<br>• ACR CIRR

	• ImmPort<br>• N3C<br>• QMENTA imaging database<br><br>Registries:<br>• SCMR COVID-19 Registry	

Supplementary Figure 4. Standards-based and technical interoperability between all of the COVID-19 data sharing resources

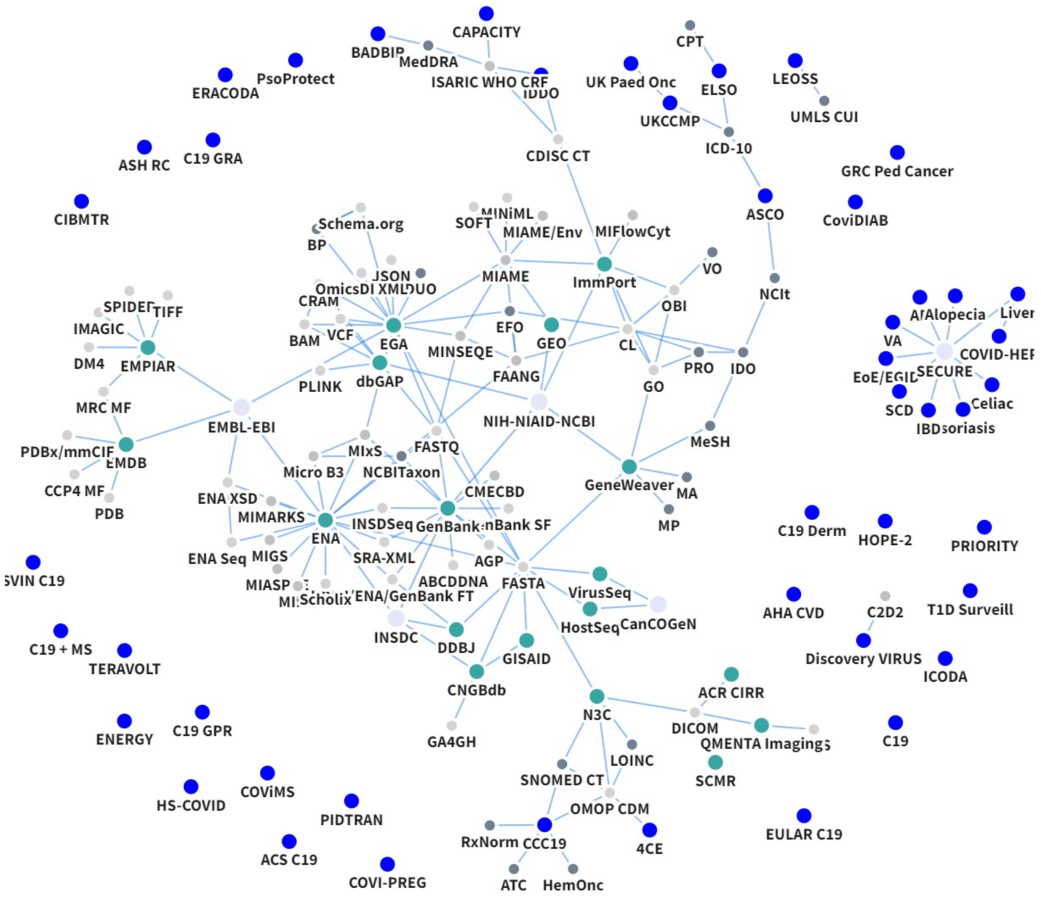

In several instances, platforms for sharing high dimensional human or pathogen omics data or imaging data accept data in any community-developed standard; the figure includes a subset of the standards used in those cases.

Supplementary Figure 5. Standards-based and technical interoperability between the COVID-19 registries

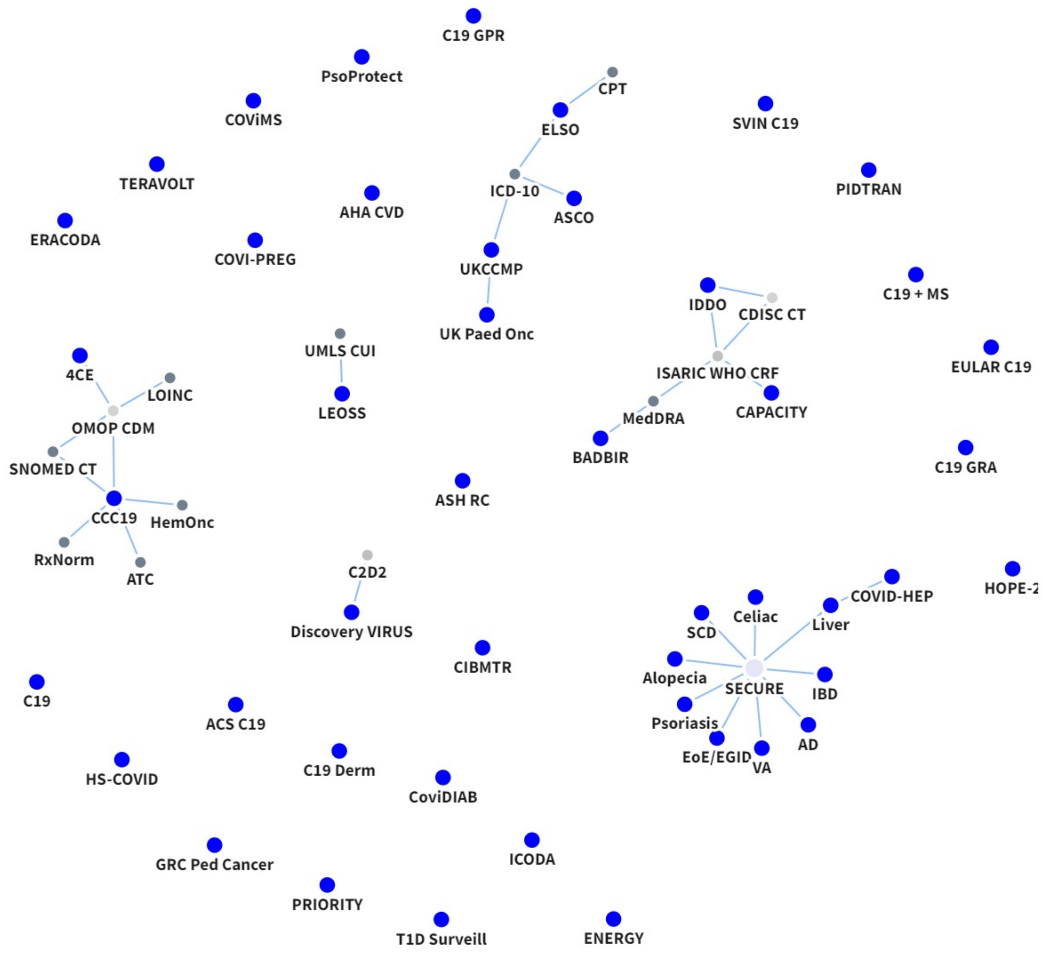

Supplementary Figure 6. Standards-based and technical interoperability between the COVID-19 platforms

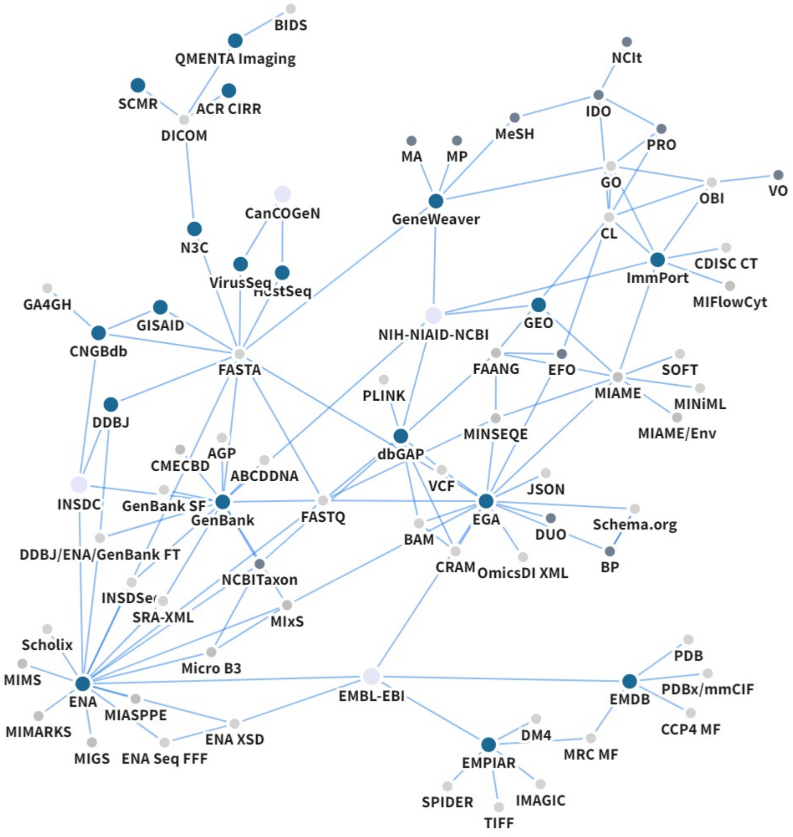

In several instances, platforms for sharing high dimensional human or pathogen omics data or imaging data accept data in any community-developed standard; the figure includes a subset of the standards used in those cases.

References

1. Dash S, Shakyawar SK, Sharma M, Kaushik S. Big data in healthcare: management, analysis and future prospects. *Journal of Big Data* 2019; **6**(1): 54.
2. Gliklich R, Dreyer N. Executive Summary. In: RE G, NA D, eds. Registries for Evaluating Patient Outcomes: A User's Guide. 2nd ed. Rockville, MD, USA: Agency for Healthcare Research and Quality; 2010.
3. King G. A Comparative Review of Various Data Repositories. July 25, 2017. https://dataverse.org/blog/comparative-review-various-data-repositories (accessed August 24, 2023).
4. Data Hub vs Data Lake vs Data Virtualization. 2021. https://www.marklogic.com/product/comparisons/data-hub-vs-data-lake/ (accessed August 24, 2023).
5. What Is a Data Warehouse? 2021. https://www.oracle.com/sg/database/what-is-a-data-warehouse/ (accessed August 24, 2023).
6. Allemang D, Hendler J. Chapter 4 - Semantic Web application architecture. In: Allemang D, Hendler J, eds. Semantic Web for the Working Ontologist (Second Edition). Boston: Morgan Kaufmann; 2011: 51-60.
7. Data Catalog. https://old.nnlm.gov/data/thesaurus/data-catalog (accessed August 24, 2023).
8. Lu Wang L, Lo K, Chandrasekhar Y, et al. CORD-19: The Covid-19 Open Research Dataset. *ArXiv* 2020: arXiv:2004.10706v2.
9. Srinivasa-Desikan B. spaCy's Language Models. Natural Language Processing and Computational Linguistics: A practical guide to text analysis with Python, Gensim, spaCy, and Keras. Birmingham, UK: Packt Publishing Ltd, 2018: 33–48.
10. Al Omran FNA, Treude C. Choosing an NLP library for analyzing software documentation: a systematic literature review and a series of experiments. 2017 IEEE/ACM 14th International Conference on Mining Software Repositories (MSR); May 20–21, 2017; Buenos Aires, Argentina.
11. Clarke DJB, Wang L, Jones A, et al. FAIRshake: Toolkit to Evaluate the FAIRness of Research Digital Resources. *Cell Systems* 2019; **9**:417–21.
12. FAIR Data Maturity Model. Specification and Guidelines (1.0). 2020. https://www.rd-alliance.org/group/fair-data-maturity-model-wg/outcomes/fair-data-maturity-model-specification-and-guidelines-0 (accessed August 24, 2023).
13. Group G-RDSW. 2018. Principles of data sharing in public health emergencies: Global Research Collaboration for Infectious Disease Preparedness (GloPID-R). https://www.glopid-r.org/wp-content/uploads/2022/07/glopid-r-principles-of-data-sharing-in-public-health-emergencies.pdf (accessed August 24, 2023).
14. COVID-19 National Core Studies Data Sharing Principles. 2021. https://www.hdruk.org/covid-19/covid-19-national-core-studies/ncs-data-sharing-principles/ (accessed August 24, 2023).
15. Knoppers BM, Harris JR, Tassé AM, et al. Towards a data sharing code of conduct for international genomic research. Genome Med 2011; **3**: 46.
16. Knoppers BM. Framework for responsible sharing of genomic and health-related data. *HUGO J* 2014; **8**:3.
17. Carroll SR, Garba I, Figueroa-Rodríguez OL, et al. The CARE Principles for Indigenous Data Governance. *Data Science Journal* 2020; **18**:43.
